# Phylogenetic diversity, trichothecene potential, and pathogenicity within *Fusarium sambucinum* species complex

**DOI:** 10.1371/journal.pone.0245037

**Published:** 2021-01-12

**Authors:** Imane Laraba, Susan P. McCormick, Martha M. Vaughan, David M. Geiser, Kerry O’Donnell

**Affiliations:** 1 USDA, Agricultural Research Service, National Center for Agricultural Utilization Research, Mycotoxin Prevention and Applied Microbiology Research Unit. 1815 N. University, Peoria, IL, United States of America; 2 Department of Plant Pathology and Environmental Microbiology, Pennsylvania State University, University Park, Pennsylvania, PA, United States of America; Georg-August-Universitat Gottingen, GERMANY

## Abstract

The *Fusarium sambucinum* species complex (FSAMSC) is one of the most taxonomically challenging groups of fusaria, comprising prominent mycotoxigenic plant pathogens and other species with various lifestyles. Among toxins produced by members of the FSAMSC, trichothecenes pose the most significant threat to public health. Herein a global collection of 171 strains, originating from diverse hosts or substrates, were selected to represent FSAMSC diversity. This strain collection was used to assess their species diversity, evaluate their potential to produce trichothecenes, and cause disease on wheat. Maximum likelihood and Bayesian analyses of a combined 3-gene dataset used to infer evolutionary relationships revealed that the 171 strains originally received as 48 species represent 74 genealogically exclusive phylogenetically distinct species distributed among six strongly supported clades: *Brachygibbosum*, *Graminearum*, *Longipes*, *Novel*, *Sambucinum*, and *Sporotrichioides*. Most of the strains produced trichothecenes in vitro but varied in type, indicating that the six clades correspond to type A, type B, or both types of trichothecene-producing lineages. Furthermore, five strains representing two putative novel species within the *Sambucinum* Clade produced two newly discovered type A trichothecenes, 15-keto NX-2 and 15-keto NX-3. Strains of the two putatively novel species together with members of the *Graminearum* Clade were aggressive toward wheat when tested for pathogenicity on heads of the susceptible cultivar Apogee. *In planta*, the *Graminearum* Clade strains produced nivalenol or deoxynivalenol and the aggressive *Sambucinum* Clade strains synthesized NX-3 and 15-keto NX-3. Other strains within the *Brachygibbosum*, *Longipes*, *Novel*, *Sambucinum*, and *Sporotrichioides* Clades were nonpathogenic or could infect the inoculated floret without spreading within the head. Moreover, most of these strains did not produce any toxin in the inoculated spikelets. These data highlight aggressiveness toward wheat appears to be influenced by the type of toxin produced and that it is not limited to members of the *Graminearum* Clade.

## Introduction

*Fusarium* contains numerous plant pathogenic and toxigenic species that pose a growing threat to a wide range of economically important crops [1]. These species are responsible for a plethora of serious diseases, which result in significant losses in crop yields and quality worldwide. Economic losses are commonly compounded by contamination of food and feed with fusarial toxins that render them unsuitable for consumption by humans and other animals [2,3]. Genealogical Concordance Phylogenetic Species Recognition (GCPSR; [4]) based analyses have resolved *Fusarium* into at least 300 phylogenetically distinct species distributed among 23 monophyletic species complexes and several single-species lineages [1 and references therein,^3^]. With 41 named species, the *F*. *sambucinum* species complex (FSAMSC) is among the most species-rich lineages within *Fusarium* [3,5–8]. This species complex comprises some of the most destructive agricultural pathogens, including members of the *F*. *graminearum* species complex and closely related species (hereafter *Graminearum* Clade; FSAMSC-1 sensu [9]), known to cause Fusarium head blight (FHB) outbreaks on small-grain cereals worldwide [9–12]. FHB epidemics have resulted in multibillion-dollar losses to the North American and global wheat industry due to significant reductions in grain yields and heavy contamination with mycotoxins [13].

Many species in the FSAMSC are known to produce a cocktail of toxic secondary metabolites in plant tissues including trichothecenes, which are among the mycotoxins of greatest concern to food safety and human health [14 and references therein]. Trichothecenes are potent inhibitors of eukaryotic protein synthesis and can cause neurologic, gastrointestinal, immunological, and other health problems [15,16]. Production of certain trichothecenes *in planta* appears to contribute to *Fusarium* pathogenicity on multiple crops where they act as virulence factors that overcome plant defenses and facilitate colonization of host tissues [17–19]. The enzymes required for trichothecene biosynthesis are encoded by *TRI* genes located at three different loci: a 12 gene core *TRI* cluster, *TRI1*―*TRI16* 2-gene cluster, and *TRI101* [20]. Functional analyses of *TRI* biosynthetic genes indicated that gain, loss, and changes in function of some genes have contributed to trichothecene structural diversity [21–24]. *Fusarium* trichothecenes are classified into two major structural types based on the absence (type A) or the presence (type B) of a carbonyl group (keto) at the C-8 position on the trichothecene ring [20]. Species within the *Graminearum* Clade, for example, have been shown to produce type B trichothecenes with one of three chemical profiles (chemotypes): 3-acetyldeoxynivalenol (3ADON), 15-acetyldeoxynivalenol (15ADON), or nivalenol (NIV) [25,26]. However, a new type A trichothecene analog similar to 3ADON but lacking the C-8 keto group named NX-2 was recently discovered in *F*. *graminearum* populations from north central U.S. and Canada [9,27,28]. Structural diversity at C-7 and C-8 results from a unique variant of the trichothecene biosynthetic *TRI1* gene product, a cytochrome P450 monooxygenase, that catalyzes C-7 but not C-8 hydroxylation [23,28]. Some other species within the FSAMSC are known to produce type A trichothecenes such as diacetoxyscirpenol (DAS), T-2 toxin (T-2), neosolaniol (NEO), and other structurally diverse type A toxins [20,29].

Application of phylogenetic species recognition based on multilocus GCPSR over the past two decades has revolutionized *Fusarium* systematics and changed our understanding of species limits, evolutionary relationships, and toxigenic potential of this agronomically important genus [3,29]. Because *F*. *graminearum* and some other species within the *Graminearum* Clade associated with cereals have been the main focus of the global scientific community over the past three decades, their ability to induce FHB and Fusarium crown rot (FCR) on cereals and produce toxins are well characterized [11,30 and references therein–32]. However, no comprehensive molecular phylogenetic study of the FSAMSC has been conducted. In addition, little is known about the potential of many species in this species complex to synthesize trichothecenes and cause FHB and FCR. Given the threat that these species pose to agricultural biosecurity, the present study was initiated to (i) collect and analyze multilocus DNA sequence data to reassess the phylogenetic diversity of 171 strains within the FSAMSC obtained from the Fusarium Research Center (FRC) at Pennsylvania State University and/or the Agricultural Research Service Culture Collection (NRRL), Peoria, Illinois, U.S.; (ii) test strains for their potential to produce trichothecenes in liquid media and on solid substrates; and (iii) evaluate their ability to induce FHB and synthesize trichothecenes in wheat.

## Materials and methods

### Strains studied

A global collection of FSAMSC strains (n = 171) selected to represent the phylogenetic diversity of this species complex was included in the present study (Table 1, S1 Table). Strains were selected based on analyses of partial translation elongation factor sequence data. Only one strain of the 23 species in the *Graminearum* Clade was included in the present study because they have been the focus of several recent studies by our research group [6, reviewed in 38]. The strains were recovered from various hosts/substrates and are available from FRC (http://plantpath.psu.edu/facilities/fusarium-research-center) and/or the ARS Culture Collection (https://nrrl.ncaur.usda.gov/) upon request.

**Table 1:** *Fusarium sambucinum* species complex strains used and toxins produced in present study and reported in the literature.

Clade^a^	Species ID^b^	Strain no.^c^	Geographic origin	Host/substrate	Toxin produced in vitro^d^	Toxin reported^e^	Reference
*Brachygibbosum*	*F*. *brachygibbosum*	FRC R-7584	Ban Tong, Thailand	Virgin jungle soil	NEO	FUS-X, 4,15-diANIV	[33,34]
*Brachygibbosum*	*F*. *brachygibbosum*	FRC R-7630	Ban Tong, Thailand	Virgin jungle soil	DAS, NEO		
*Brachygibbosum*	*F*. *brachygibbosum*	FRC R-7637	Ban Tong, Thailand	Virgin jungle soil	DAS, NEO		
*Brachygibbosum*	*F*. *brachygibbosum*	FRC R-8851	Kolo, Niger	Pearl millet	DAS, NEO		
*Brachygibbosum*	*F*. *brachygibbosum*	NRRL 20954	India	Sorghum	―		
*Brachygibbosum*	*F*. sp. nov.-23	FRC R-4948	Iran	Citrus root	DAS, NEO	BEA, DAS, 8-ANEO	[29]
*Brachygibbosum*	*F*. sp. nov.-23	FRC R-7475	Ban Pha Baen, Thailand	Banana grove soil	DAS, NEO		
*Brachygibbosum*	*F*. sp. nov.-23	FRC R-7513	TX, USA	Sorghum soil debris	―		
*Brachygibbosum*	*F*. sp. nov.-23	FRC R-7610	MS, USA	Sorghum soil debris	DAS, NEO		
*Brachygibbosum*	*F*. sp. nov.-23	FRC R-7868	AZ, USA	Guayule crown	DAS, NEO		
*Brachygibbosum*	*F*. sp. nov.-23	FRC R-8117	Tunis, Tunisia	Soil	DAS, NEO		
*Brachygibbosum*	*F*. sp. nov.-23	FRC R-8321	TX, USA	Peanut soil debris	DAS, NEO		
*Brachygibbosum*	*F*. sp. nov.-23	FRC R-8846	CA, USA	Human nasal sore	―		
*Brachygibbosum*	*F*. sp. nov.-23	FRC R-8876	Beijing, China	Rice kernel	NEO		
*Brachygibbosum*	*F*. sp. nov.-23	NRRL 13829	Japan	River sediment	DAS, NEO		
*Brachygibbosum*	*F*. sp. nov.-23	NRRL 28448	Turkey	Banana	NEO		
*Brachygibbosum*	*F*. sp. nov.-24	FRC R-9118	China	Soil	DAS, NEO	NR	
*Brachygibbosum*	*F*. sp. nov.-24	NRRL 66936	China	Soil	CAL, DAS, NEO		
*Brachygibbosum*	*F*. sp. nov.-25	FRC R-8124	Mahdes, Tunisia	Olive grove soil	NEO	NR	
*Brachygibbosum*	*F*. sp. nov.-25	NRRL 66928	Mahdes, Tunisia	Olive grove soil	DAS, NEO		
*Brachygibbosum*	*F*. sp. nov.-25	NRRL 66931	Italy	Unknown	NEO		
*Brachygibbosum*	*F*. sp. nov.-26	FRC R-8856	CA, USA	Melon root	―	NR	
*Brachygibbosum*	*F*. sp. nov.-26	NRRL 66923	ND, USA	Pasture soil debris	DAS, NEO		
*Brachygibbosum*	*F*. sp. nov.-27	FRC R-7642	Puerto Rico	Sorghum debris	―	NR	
*Brachygibbosum*	*F*. sp. nov.-27	FRC R-8881	Nigeria	Unknown	NEO		
*Brachygibbosum*	*F*. sp. nov.-27	FRC R-9025	Mashonaland Central, Zimbabwe	Millet soil debris	NEO		
*Brachygibbosum*	*F*. sp. nov.-27	KOD 1825	GA, USA	Peanuts	NEO		
*Brachygibbosum*	*F*. sp. nov.-27	NRRL 66930	IL, USA	Corn soil	NEO		
*Brachygibbosum*	*F*. sp. nov.-28	FRC R-9121	China	Soil	DAS, NEO	NR	
*Brachygibbosum*	*F*. sp. nov.-28	NRRL 66939	Anda, China	Soybean root	DAS, NEO		
*Brachygibbosum*	*F*. sp. nov.-29	FRC R-8779	GA, USA	Sorghum soil debris	―	4,15-diANIV	[34]
*Brachygibbosum*	*F*. sp. nov.-29	FRC R-9012	Zaria, Kaduna, Nigeria	Soil	NIV		
*Brachygibbosum*	*F*. sp. nov.-29	KOD 1715	Ghana	Soybean roots	NIV		
*Brachygibbosum*	*F*. sp. nov.-29	KOD 1726	Ghana	Soybean roots	NIV		
*Brachygibbosum*	*F*. sp. nov.-29	NRRL 28725	Argentina	Wheat	―		
*Brachygibbosum*	*F*. sp. nov.-29	NRRL 66920	Ghana	Soybean roots	NIV		
*Brachygibbosum*	*F*. sp. nov.-29	NRRL 66922	Brazil	Wheat sub-crown internode	DAS, NIV		
*Brachygibbosum*	*F*. sp. nov.-30	FRC R-8886	Nigeria	Unknown	―	NR	
*Brachygibbosum*	*F*. sp. nov.-30	NRRL 66937	Pandamatenga, Botswana	Pearl millet	NIV		
*Brachygibbosum*	*F*. sp. nov.-31	NRRL 66919	Ghana	Soybean roots	DAS, NIV	4,15-diANIV	[34]
*Brachygibbosum*	*F*. sp. nov.-32	FRC R-6436	Tarlac, Philippines	Grassland soil	―	NR	
*Brachygibbosum*	*F*. sp. nov.-32	NRRL 66938	Nakhon Sawan, Thailand	Corn	DAS		
*Brachygibbosum*	*F*. sp. nov.-33	FRC R-9125	Australia	Soil	―	NR	
*Brachygibbosum*	*F*. sp. nov.-33	NRRL 66933	NT, Australia	Cultivated soil	DAS, NIV		
*Brachygibbosum*	*F*. *transvaalense*	FRC R-4478	Australia	Soil	BUT	4,15-diANIV	[34]
*Brachygibbosum*	*F*. *transvaalense*	FRC R-6827	Zululand, South Africa	Natural vegetation debris	DAS, NIV		
*Brachygibbosum*	*F*. *transvaalense*	FRC R-6855	Zululand, South Africa	Natural vegetation debris	―		
*Brachygibbosum*	*F*. *transvaalense*	FRC R-7052	PA, USA	Oat horse feed	―		
*Brachygibbosum*	*F*. *transvaalense*	NRRL 31008	Australia	Soil	―		
*Brachygibbosum*	*F*. *transvaalense*	NRRL 32021	Australia	Unknown	―		
*Graminearum*	*F*. *acaciae-mearnsii*	NRRL 26755	South Africa	Australian acacia	CUL, NIV	DON, NIV, 3ADON, 4-ANIV	[10,35]
*Graminearum*	*F*. *aethiopicum*	NRRL 46726	Amhara region, Ethiopia	Wheat seed	CUL, 15ADON	DON, 15ADON	[36]
*Graminearum*	*F*. *asiaticum*	NRRL 13818	Japan	Barley	―	BUT, DON, NIV, ZEA, 3ADON, 4-ANIV, 4,15-diANIV, 15ADON	[10,29,37]
*Graminearum*	*F*. *austroamericanum*	NRRL 28585	Brazil	Herbaceous vine	NIV	DON, NIV, ZEA, 3ADON, 4-ANIV	[10]
*Graminearum*	*F*. *boothii*	NRRL 26916	South Africa	Corn	CUL, 3ADON	BUT, CUL, DON, ZEA, 15ADON	[10,29,37]
*Graminearum*	*F*. *brasilicum*	NRRL 31238	Pelotas, RS, Brazil	Barley	CAL, CUL, NIV	NIV, 3ADON	[38]
*Graminearum*	*F*. *cerealis*	NRRL 13721	Posnan, Poland	Potato	NIV	NIV, ZEA, 4-ANIV, 4,15-diANIV	[10,29]
*Graminearum*	*F*. *cortaderiae*	NRRL 29297	New Zealand	Toetoe (*Cortaderia* sp.)	CUL, NIV	NIV, 15ADON	[26,39]
*Graminearum*	*F*. *culmorum*	NRRL 25475	Denmark	Barley kernel	―	BIK, BUT, CUL, NIV, OHCUL, ZEA, 3ADON, 4,15-diANIV, 15ADON	[10,29,32,40]
*Graminearum*	*F*. *dactylidis*	NRRL 29380	USA	Unknown	―	NIV, ZEA	[41]
*Graminearum*	*F*. *gerlachii*	NRRL 36905	MN, USA	Wheat	CUL, NIV	NIV	[35]
*Graminearum*	*F*. *graminearum*	NRRL 31084	MI, USA	Corn	CUL, 15ADON	BEA, BUT, CUL, DON, NIV, OHCUL, ZEA, 3ADON, 4-ANIV, 15ADON	[10,29,35,42]
*Graminearum*	*F*. *louisianense*	NRRL 54197	LA, USA	Unknown	―	NIV	[43]
*Graminearum*	*F*. *lunulosporum*	NRRL 13393	South Africa	Grapefruit	ZEA	NR	
*Graminearum*	*F*. *meridionale*	NRRL 28436	New Caledonia	Sweet potato	NIV	NIV, ZEA, 4-ANIV	[10]
*Graminearum*	*F*. *mesoamericanum*	NRRL 25797	Honduras	Banana	―	DON, ZEA	[10]
*Graminearum*	*F*. *nepalense*	NRRL 54222	Nepal	Unknown	CUL, 15ADON	DON, 15ADON	[43]
*Graminearum*	*F*. *praegraminearum*	NRRL 39664	Wellington, New Zealand	Litter in corn field	CUL, NIV	CUL, OHCUL, ZEA, 4-ANIV, 4,15-diANIV	[5]
*Graminearum*	*F*. *pseudograminearum*	NRRL 28062	New South Wales, Australia	Barley crown	CUL, 3ADON	DON, NIV, ZEA, 3ADON	[10,26,39]
*Graminearum*	*F*. sp. nov.-5	NRRL 34461	Umyaka, South Africa	Soil	CUL, 3ADON	DON, 3ADON	[35]
*Graminearum*	*F*. *subtropicale*	NRRL 66764	Paraná, Brazil	Barley	CUL, NIV	BUT, CUL, FUS C, 4,15-diANIV	[6]
*Graminearum*	*F*. *ussurianum*	NRRL 45681	Primorsky Krai, Russia	Oat	CUL, 3ADON	DON, 3ADON	[44]
*Graminearum*	*F*. *vorosii*	NRRL 37605	Hungary	Unknown	CUL, 15ADON	DON, NIV, ZEA, 3ADON, 4-ANIV, 15ADON	[35,37]
*Longipes*	*F*. *longipes*-1	FRC R-3460	Australia	Soil	―	BEA	[45]
*Longipes*	*F*. *longipes*-1	NRRL 13368	Australia	Soil	DAS		
*Longipes*	*F*. *longipes*-2	FRC R-5122	New Guinea	Pasture soil	―	NR	
*Longipes*	*F*. *longipes*-2	FRC R-5127	New Guinea	Soil debris	―		
*Longipes*	*F*. *longipes*-2	NRRL 13374	New Guinea	Soil debris	―		
*Longipes*	*F*. *longipes*-3	NRRL 20723	United Kingdom	Unknown	―	NR	
*Longipes*	*F*. *longipes*-4	FRC R-7358	Guanxi, China	Weedy streamside soil	―	NR	
*Longipes*	*F*. *longipes*-4	FRC R-7602	Thailand	Pasture soil debris	―		
*Longipes*	*F*. *longipes*-4	FRC R-7695	Ban Than, Thailand	Soil	―		
*Longipes*	*F*. *longipes*-4	FRC R-8789	Philippines	Soil	―		
*Longipes*	*F*. *longipes*-4	NRRL 13317	CA, USA	Unknown	―		
*Longipes*	*F*. *longipes*-4	NRRL 20695	FL, USA	Soil	DAS		
*Longipes*	*F*. *longipes*-4	NRRL 39691	Unknown	Unknown	―		
*Longipes*	*F*. sp. nov.-18	FRC R-6894	Rockhampton, Australia	Air plate	―	NR	
*Longipes*	*F*. sp. nov.-18	NRRL 66924	Rockhampton, Australia	Soil debris	―		
*Longipes*	*F*. sp. nov.-19	NRRL 66932	NT, Australia	Grassland soil	―	NR	
*Longipes*	*F*. sp. nov.-20	NRRL 66926	Uban, Thailand	Rice paddy soil	DAS	NR	
*Longipes*	*F*. sp. nov.-21	NRRL 66935	Darwin, Australia	Soil debris	DAS	NR	
*Longipes*	*F*. sp. nov.-22	NRRL 66934	Niger State, Nigeria	Sorghum soil debris	―	NR	
*Novel*	*F*. sp. nov.-6	NRRL 28066	Japan	Corn	T-2	BEA, T-2	[29]
*Novel*	*F*. sp. nov.-6	NRRL 54640	Papua New Guinea	Sweet potato stem	NEO		
*Novel*	*F*. sp. nov.-6	NRRL 54683	Papua New Guinea	Sweet potato stem	DAS, NEO, T-2		
*Novel*	*F*. sp. nov.-7	NRRL 46743	Ethiopia	Wheat seed	NEO, T-2	T-2	[34]
*Novel*	*F*. sp. nov.-7	NRRL 66736	Ethiopia	Soybean roots	NEO, T-2		
*Sambucinum*	*F*. *kyushuense*	NRRL 3509	Kyushu, Japan	Wheat seed	DAS, NIV	BEA, BUT, diNIV, ENB, ENB_1_, FUS-X, NIV, 4,15-diANIV	[29,46–48]
*Sambucinum*	*F*. *kyushuense*	NRRL 6491	Kochi, Shikoku, Japan	Vinyl plate	DAS, NIV		
*Sambucinum*	*F*. *musarum*	NRRL 28507	Panama	Banana	T-2	DAS, HT-2, NEO, T-2	[49]
*Sambucinum*	*F*. *poae*	NRRL 25799	Germany	Sweet vernal grass	DAS	BEA, BUT, CUL, DAS, ENA_1_, ENB, ENB_1_, FUS-X, NEO, NIV, SCR, 15-MAS	[48,50]
				(*Anthoxanthum odoratum*)			
*Sambucinum*	*F*. *poae*	NRRL 31280	Brazil	Oat (var. Linhagem 469)	―		
*Sambucinum*	*F*. *poae*	NRRL 36285	Aas, Norway	Barley	DAS		
*Sambucinum*	*F*. *robustum*	NRRL 13392	Argentina	Paraná pine (*Araucaria angustifolia*)	NEO, T-2	NR	
*Sambucinum*	*F*. *sambucinum*	FRC R-4712	Switzerland	Potato tuber	DAS	BEA, DAS, ENB, NEO, T-2	[29,51,52]
*Sambucinum*	*F*. *sambucinum*	FRC R-738	NY, USA	Potato	DAS		
*Sambucinum*	*F*. *sambucinum*	NRRL 20666	United Kingdom	Potato	DAS		
*Sambucinum*	*F*. *sambucinum*	NRRL 13394	New Zealand	Lupine (*Lupinus* sp.)	DAS, NEO, T-2		
*Sambucinum*	*F*. *sambucinum*	NRRL 20663	Germany	Unknown	DAS		
*Sambucinum*	*F*. *sambucinum*	NRRL 31964	New Zealand	Scotch broom (*Cytisus scoparius*)	DAS, NEO		
*Sambucinum*	*F*. *sambucinum*	NRRL 31969	New Zealand	Gorse (*Ulex europaeus*)	T-2		
*Sambucinum*	*F*. sp. nov.-17	NRRL 26795	USA	Soil	BUT, CAL, DAS	NR	
*Sambucinum*	*F*. sp. nov.-10	FRC R-7403	South Africa	Potato	T-2	NEO, T-2	[53]
*Sambucinum*	*F*. sp. nov.-10	NRRL 22189	Brazil	Soybean	NEO, T-2		
*Sambucinum*	*F*. sp. nov.-11	NRRL 22192	Indonesia	Palm tree	―	T-2	[54]
*Sambucinum*	*F*. sp. nov.-12	FRC R-642	Unknown	Carnation ‘Iroquois’	DAS	NR	
*Sambucinum*	*F*. sp. nov.-12	FRC R-7107	Transkei, South Africa	Soil	DAS		
*Sambucinum*	*F*. sp. nov.-12	FRC R-7122	Transkei, South Africa	Soil	DAS, 3OH		
*Sambucinum*	*F*. sp. nov.-12	FRC R-8227	Zinggayi, South Africa	Debris	DAS		
*Sambucinum*	*F*. sp. nov.-12	NRRL 66929	Zinggayi, South Africa	Debris	DAS		
*Sambucinum*	*F*. sp. nov.-13	NRRL 39635	Taupo, New Zealand	Toetoe (*Cortaderia* sp.)	DAS	NR	
*Sambucinum*	*F*. sp. nov.-13	NRRL 39685	Gisborne, New Zealand	Toetoe (*Cortaderia* sp.)	BUT, CUL, DAS		
*Sambucinum*	*F*. sp. nov.-14	NRRL 36134	Unknown	Unknown	DAS, NEO, T-2	NR	
*Sambucinum*	*F*. sp. nov.-15	FRC R-8154	Transkei, South Africa	Soil	CUL, 3,7-diOH, 15-decal, 15-keto NX-2/NX-3, 15-OHCUL	NR	
*Sambucinum*	*F*. sp. nov.-15	FRC R-8203	Transkei, South Africa	Debris			
*Sambucinum*	*F*. sp. nov.-15	NRRL 66921	South Africa	Wheat straw			
*Sambucinum*	*F*. sp. nov.-16	FRC R-8136	Transkei, South Africa	Debris	CUL, 3,7-diOH, 15-decal, 15-keto NX-2/NX-3, 15-OHCUL	NR	
*Sambucinum*	*F*. sp. nov.-16	NRRL 66927	South Africa	Natural vegetation soil			
*Sambucinum*	*F*. sp. nov.-8	NRRL 29296	Auckland, New Zealand	Chayote (*Sechium edule*)	DAS, NEO, T-2	NR	
*Sambucinum*	*F*. sp. nov.-9	NRRL 13465	Argentina	Tiger pear (*Opuntia aurantica*)	T-2	NR	
*Sambucinum*	*F*. sp. nov.-9	NRRL 66925	Argentina	Tiger pear (*Opuntia aurantica*)	DAS, T-2		
*Sambucinum*	*F*. *venenatum*	FRC R-2273	Australia	Corn	DAS	BEA, BUT, CUL, DAS, SCR, 4-MAS, 15-ASCR	[29,55,56]
*Sambucinum*	*F*. *venenatum*	FRC R-8978	Ordu, Turkey	Soil	BUT, DAS		
*Sambucinum*	*F*. *venenatum*	NRRL 22196	Germany	Corn	DAS		
*Sambucinum*	*F*. *venenatum*	NRRL 22198	Finland	Unknown	BUT, DAS, 3OH		
*Sambucinum*	*F*. *venenatum*	NRRL 25413	England	Unknown	DAS		
*Sambucinum*	*F*. *venenatum*	NRRL 26228	Austria	Wheat	DAS		
*Sambucinum*	*F*. *venenatum*	NRRL 31989	Australia	Soil	BUT, CAL, DAS		
*Sambucinum*	*F*. *venenatum*	NRRL 32015	Australia	Soil	BUT, CAL, DAS		
*Sporotrichioides*	*F*. *armeniacum*	FRC R-6472	FL, USA	Slash pine seedling	NEO, T-2	BEA, DAS, HT-2, MAS, NEO, T-2, T-2TE, T-2TR, ZEA	[57]
*Sporotrichioides*	*F*. *armeniacum*	FRC R-7479	Australia	Sorghum	DAS, T-2		
*Sporotrichioides*	*F*. *armeniacum*	FRC R-8519	GA, USA	Corn soil	DAS, NEO, T-2		
*Sporotrichioides*	*F*. *armeniacum*	FRC R-8609	Abetshawe, South Africa	Soil debris	DAS, NEO, T-2		
*Sporotrichioides*	*F*. *armeniacum*	NRRL 13343	Unknown	Unknown	DAS, NEO, T-2		
*Sporotrichioides*	*F*. *goolgardii*	KOD 1087	Bungonia, Australia	Grass tree (*Xanthorrhoea glauca)*	DAS, NEO, T-2	DAS, NEO, T-2	[58]
*Sporotrichioides*	*F*. *goolgardii*	KOD 1090	Bungonia, Australia	Grass tree (*Xanthorrhoea glauca)*	DAS, NEO, T-2		
*Sporotrichioides*	*F*. *langsethiae*	NRRL 34176	Unknown	Unknown	CUL, DAS, NEO, T-2	BEA, CUL, DAS, ENA_1_, ENB, ENB_1_, HT-2, iso-NEO, NEO, OHCUL, T-2, T-2TE, T-2TR	[48,59,60]
*Sporotrichioides*	*F*. *langsethiae*	NRRL 36236	Unknown	Unknown	CUL, DAS, NEO, T-2		
*Sporotrichioides*	*F*. *langsethiae*	NRRL 54940	Norway	Oat	NEO		
*Sporotrichioides*	*F*. *nodosum*	NRRL 13431	Turkey	Tomato stem	―	NR	
*Sporotrichioides*	*F*. *nodosum*	NRRL 36351	Lisboa, Portugal	Stored peanuts	DAS		
*Sporotrichioides*	*F*. *palustre*	NRRL 43289	Unknown	Spartina rhizosphere soil	DAS, NEO, T-2	DAS, NEO, T-2, 8-ANEO	[58]
*Sporotrichioides*	*F*. *palustre*	NRRL 54056	CT, USA	Smooth cordgrass (*Spartina alterniflora)*	DAS, T-2		
*Sporotrichioides*	*F*. *palustre*	NRRL 54058	CT, USA	Smooth cordgrass (*Spartina alterniflora)*	DAS, T-2		
*Sporotrichioides*	*F*. *sibiricum*	NRRL 53429	Vladivostok, Russia	Oat	T-2	DAS, T-2	[60]
*Sporotrichioides*	*F*. *sibiricum*	NRRL 53430	Chabarovsk, Russia	Oat	DAS, NEO, T-2		
*Sporotrichioides*	*F*. sp. nov.-1	FRC R-3766	Australia	Soil	DAS, T-2	NR	
*Sporotrichioides*	*F*. sp. nov.-1	FRC R-8102	Free State, South Africa	Oat	DAS, NEO, 3OH		
*Sporotrichioides*	*F*. sp. nov.-2	FRC R-5100	Australia	Corn	DAS, T-2	NR	
*Sporotrichioides*	*F*. sp. nov.-2	NRRL 54062	CT, USA	Smooth cordgrass (*Spartina alterniflora)*	DAS, NEO, T-2		
*Sporotrichioides*	*F*. sp. nov.-3	FRC R-8090	South Africa	Oat	NEO, T-2	NR	
*Sporotrichioides*	*F*. sp. nov.-3	FRC R-9068	Manitoba, Canada	Corn seed	T-2		
*Sporotrichioides*	*F*. sp. nov.-4	NRRL 29896	Netherlands	Unknown	DAS	NR	
*Sporotrichioides*	*F*. sp. nov.-4	NRRL 29897	Unknown	Unknown	―		
*Sporotrichioides*	*F*. *sporotrichioides*	NRRL 13440	WI, USA	Grain elevator	DAS, NEO, T-2	BEA, BUT, CUL, DAS, ENA_1_, ENB, ENB_1_, FUS C, HT-2, NEO, SCR, T-2, T-2TE, T-2TR, 15-MAS	[42,48,51,61]
*Sporotrichioides*	*F*. *sporotrichioides*	NRRL 25474	Germany	Norway spruce (*Picea abies*) seed	DAS, NEO, T-2		
*Sporotrichioides*	*F*. *sporotrichioides*	NRRL 26923	Japan	Peas	DAS, NEO, T-2		
*Sporotrichioides*	*F*. *sporotrichioides*	NRRL 29131	Germany	Oat kernel	DAS, NEO, T-2		
*Sporotrichioides*	*F*. *sporotrichioides*	NRRL 3299	France	Corn	DAS, T-2		
*Sporotrichioides*	*F*. *sporotrichioides*	NRRL 36295	Heverlee, Belgium	Tobacco seedling root	DAS, NEO, T-2		
*Sporotrichioides*	*F*. *sporotrichioides*	NRRL 52731	Unknown	Unknown	DAS, NEO, T-2		

^a^*Brachygibbosum*, *Fusarium brachygibbosum* Clade; *Graminearum*, *F*. *graminearum* Clade; *Longipes*, *F*. *longipes* Clade; *Novel*, *Novel* Clade; *Sambucinum*, *F*. *sambucinum* Clade; *Sporotrichioides*, *F*. *sporotrichioides* Clade.

^b^*F*. sp. nov.-1 to -33, putatively novel species resolved within the FSAMSC; *F*. *longipes*-1 to-4, four phylogenetically distinct species within the *F*. *longipes* Clade previously reported in other studies.

^c^FRC, Fusarium Research Center, The Pennsylvania State University, University Park, Pennsylvania; NRRL, ARS Culture Collection, Peoria, Illinois; KOD, available from Kerry O’Donnell.

^d^Toxins were determined via gas chromatography-mass spectrometry. BUT, butenolide; CAL, calonectrin; CUL, culmorin; DAS, diacetoxyscirpenol; NEO, neosolaniol; NIV, nivalenol; T-2, T-2 toxin; ZEA, zearalenone; 3ADON, 3-acetyldeoxynivalenol; 3,7-diOH, 7-hydroxy isotrichodermol; 3OH, isotrichodermol; 15ADON, 15-acetyldeoxynivalenol; 15-decal, 15-decalonectrin; 15-keto NX-2 and 15-keto NX-3, novel trichothecenes; 15-OHCUL; 15-hydroxy culmorin;―, toxin not detected.

^e^Toxins reported in the literature. BEA, beauvericin; BIK, bikaverin; BUT, butenolide; CUL, culmorin; DAS, diacetoxyscirpenol; DON, deoxynivalenol; ENA_1_, B, B_1_, enniatins A_1_, B, B_1_; FUS C, fusarin C; FUS-X, fusarenon X; iso-NEO, iso-neosolaniol; HT-2, HT-2 toxin; NEO, neosolaniol; NIV, nivalenol; OHCUL, hydroxy culmorin; SCR, scirpentriol; T-2, T-2 toxin; T-2TE, T-2 tetraol; T-2TR, T-2 triol; ZEA, zearalenone; 3ADON, 3-acetyldeoxynivalenol; 4-ANIV, 4-acetylnivalenol; 4,15-diANIV, 4,15-diacetylnivalenol; 4-MAS, 4-monoacetoxyscirpenol; 8-ANEO, 8-acetylneosolaniol; 15ADON, 15-acetyldeoxynivalenol; 15-ASCR, 15-acetoxyscirpenol; 15-AT-2TE, 15 acetyl T-2 tetraol; 15-MAS, 15-monoacetoxyscirpenol; NR, no reports.

### Molecular phylogenetic analysis

To obtain mycelium for DNA extraction, strains were grown in 50 mL disposable polypropylene tubes containing 25 mL yeast-malt broth (3 g yeast extract, 3 g malt extract, 5 g peptone, and 20 g dextrose per L; Difco, Detroit, Michigan) on a rotary shaker set at 200 rpm and 25 ˚C. After 3–4 days mycelia were harvested on #1 Whatman filter paper discs over a Büchner funnel, lyophilized, and ground to a fine powder. Genomic DNA was then extracted following a cetyl trimethyl-ammonium bromide method [62]. For polymerase chain reaction (PCR) amplification, total genomic DNA was diluted 1:50 in sterile deionized water in 96-well plates and stored at -20 ˚C when not in use. To assess phylogenetic diversity of the 171 strains, portions of DNA-directed RNA polymerase II largest subunit (*RPB1*), RNA polymerase second- largest subunit (*RPB2*), and translation elongation factor (*TEF1*) were amplified and then sequenced as previously described [63]. PCR amplifications were performed using Platinum *Taq* DNA polymerase (Invitrogen Life Technologies, Carlsbad, California) in an Applied Biosystems ProFlex thermocycler (ABI, Emeryville, California). Amplicons were purified using ExoSAP-IT (ABI) and Sanger sequenced using ABI BigDye v3.1 Terminator reaction mix in a ProFlex thermocycler. Sequencing reactions were then purified using ABI BigDye XTerminator and sequences were obtained using an ABI 3730 48-capillary DNA analyzer. Sequence chromatograms were edited using Sequencher 5.2.4 (Gene Codes Corp., Ann Arbor, Michigan). Sequences were then aligned with MUSCLE [64] in SeaView 4.7 [65] and individual trees were inferred from the *RPB1*, *RPB2*, and *TEF1* data sets using maximum likelihood (ML) as implemented in IQ-TREE 1.6.12 [66]. Sequences of *F*. *nelsonii* NRRL 13338 from the *F*. *chlamydosporum* species complex (FCSC) were selected as the outgroup based on more inclusive analyses [67]. Using the Bayesian information criterion scores, ModelFinder [68] identified TIMe+I+G4, TNe+G4, and TIM2e+G4, respectively, as the best-fit models of molecular evolution for the *RPB1*, *RPB2*, and *TEF1* partitions [69]. Clade support assessed by 5000 bootstrap (BS) pseudoreplicates of the data did not reveal any conflict between strongly supported nodes. Therefore, the *RPB1*, *RPB2*, and *TEF1* partitions were analyzed as a combined data set with IQ-TREE. Clade support was also assessed by Bayesian posterior probabilities (BPP). Bayesian inference (BI) analyses of the individual partitions and combined 3-locus data set were performed using MrBayes 3.2.7a [70,71] on the CIPRES Scientific Gateway (https://www.phylo.org). Generalized Time Reversible (GTR) with a gamma distribution was selected as the model for nucleotide substitution. Two runs of four MCMC (Markov Chain Monte Carlo) chains were run simultaneously for 10^7^ generations with trees sampled every 1000 generations. For each run, the first 25% trees were discarded as the burn-in phase and BPP were determined from the remaining trees. Lineages that were represented by two or more strains were considered to be phylogenetically distinct if they were supported as genealogically exclusive by a BPP ≥ 0.99 and ML bootstrap (ML-BS) ≥ 95% from one or more of the individual partitions and/or the combined data set, and none of the individual partitions contradicted their monophyly. The *RPB1*, *RPB2*, and *TEF1* sequences were deposited in GenBank NCBI (accession numbers MW233055–MW233569).

### Trichothecene production in broth, rice, and corn substrates

Two liquid culture media were used to test the ability of 171 strains to produce trichothecenes. Strains were initially grown on V8 agar [72] for 7 days at 25 ˚C. Two 0.5 cm^2^ mycelial plugs cut from V8 cultures were inoculated into 20 mL of agmatine broth [73] and 20 mL of yeast extract peptone dextrose medium (YEPD: 1 g yeast extract, 1 g peptone, and 50 g dextrose per 1 L distilled water) in 50 mL flasks. Flasks were incubated at 28 ˚C in the dark on a rotary shaker at 200 rpm. After 7 days the cultures were extracted with 8 mL of ethyl acetate. The extracts were dried under a nitrogen stream in 1-dram vials and then resuspended in 1 mL ethyl acetate. Strains that failed to synthesize trichothecenes in agmatine and YEPD broth, and some isolates of special interest, were cultured on autoclaved rice (4.4 g rice grain + 1.8 mL water). After 15 days incubation in the dark at 25 ˚C, each rice culture was extracted with 10 mL of ethyl acetate, dried under nitrogen, and then resuspended in 1 mL ethyl acetate. Strains that did not produce detectable levels of trichothecenes in rice cultures were grown on autoclaved cornmeal (4.4 g commercial Quaker^®^ cornmeal + 1.8 mL water) for 15 days in the dark at 25 ˚C. Toxins were extracted following the protocol described above. Gas chromatography-mass spectrometry (GC-MS) analyses of the ethyl acetate extracts were performed on an Agilent 6890 gas chromatograph fitted with a HP-5MS column (30 m, 0.25 mm, 0.25 μm) coupled to a 5973-mass spectrometer (Agilent, Santa Clara, California). Helium was used as the carrier gas with a 20:1 split ratio and a 20 mL/min split flow. The column was held at 150 ˚C for 1 min following injection, heated to 280 ˚C at 30 ˚C/min where it was held for 7.7 min. Compound identification was based on comparisons of their retention times and mass spectra with a library of mycotoxin standards and a NIST reference library.

### Pathogenicity test on wheat

Pathogenicity of 75 strains chosen to represent the full range of FSAMSC diversity was tested on the full-dwarf hard red spring wheat cultivar Apogee (*Triticum aestivum*) [74]. This cultivar is characterized by high susceptibility to FHB [75]. Five seeds of Apogee were planted in 2.5 L plastic pots filled with Sun Gro® Horticulture potting mix (Agawam, Massachusetts) supplemented with 2 g of Micromax, and 13 g Osmocote 14-14-14 controlled-release fertilizer (ICL Specialty Fertilizers, Summerville, South Carolina). Pots were arranged randomly and maintained in a controlled growth room under the following conditions: 50–60% relative humidity, 14 h day period, and temperature set at 23 ˚C day and 20 ˚C night. Every two weeks each pot was fertilized with 500 mL of a solution containing 0.5 g per L of Peters 20-20-20 (Grace-Sierra Horticultural Products, Milpitas, California). At anthesis, five wheat heads representing biological replicates were inoculated with one of the 75 strains by injecting a spikelet with 10 μL of 0.04% sterile Tween 20 containing 10^5^ conidia. The negative control was inoculated with 10 μL of 0.04% sterile Tween 20. *Fusarium graminearum* NRRL 31084 (= PH1), which is a well-characterized FHB pathogen [11], was used as the positive control. The number of symptomatic florets displaying premature whitening or necrosis, typical symptoms of FHB, was scored at 7, 10, 14, 17, and 21 days after inoculation. Isolates that only induced FHB symptoms in the inoculated spikelet were considered as pathogenic. Strains that overcame the plant’s defenses and spread to other florets were scored as aggressive. Disease spread was then assessed by calculating the total percentage of diseased florets 21 days post inoculation.

### Trichothecene production *in planta*

To evaluate trichothecene production *in planta*, independent of disease spread, the five wheat heads inoculated with one of the 75 strains were harvested 21 days post inoculation, combined, and frozen at -80 ˚C. These samples were freeze-dried for 48 hr and then ground to a fine powder using a Geno/Grinder 2010 automated tissue homogenizer (OPS Diagnostics, Lebanon, New Jersey). One g of each sample was extracted with 10 mL of acetonitrile-water (86:14, v/v) by shaking for 15 min. After centrifugation 5 mL of the extract were cleaned using a MycoSep 225 Trich column (Romer Labs, Union, Missouri). Two mL of the purified extract were transferred to a 1-dram vial, dried under a nitrogen stream, and then derivatized with 100 μL of a 100:1 freshly prepared mixture of N-trimethylsilylimadazole/trimethylchlorosilane (Sigma-Aldrich, St. Louis, Missouri). After 20 min, 900 μL of isooctane were added to the vial, followed by 1 mL of water. The samples were vortexed until the top organic layer became clear and then it was analyzed by GC-MS as described above.

## Results

### Molecular phylogenetic analysis

**Fig 1:**
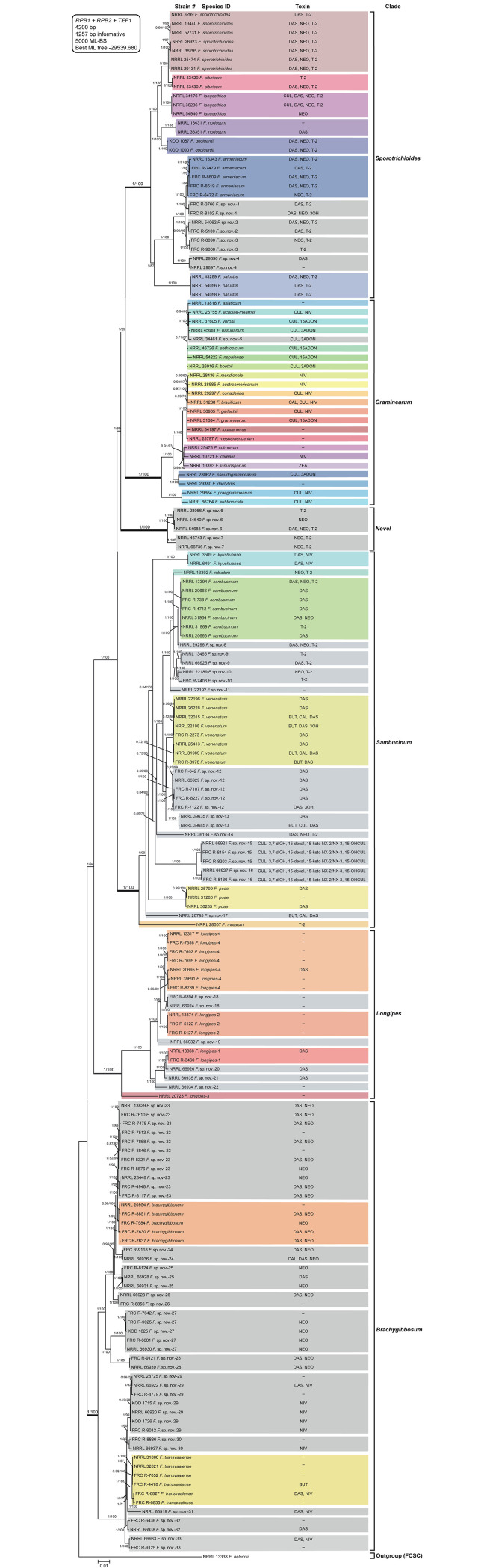
Bayesian and maximum likelihood phylogeny inferred from partial *RPB1* + *RPB2* + *TEF1* data set. Sequence data set was generated from 171 strains representing 74 species in the *Fusarium sambucinum* species complex (FSAMSC). Support values above or below branches are Bayesian posterior probabilities (BPP)/maximum likelihood bootstrap (ML-BS) values. Bayesian posterior probabilities were calculated using MrBayes 3.2.7a [71]. ML-BS values were determined using IQ-TREE 1.6.12 [66]. The FSAMSC was rooted on sequences of NRRL 13338 *F*. *nelsonii* from its sister group, the *F*. *chlamydosporum* species complex. The following six clades (from top to bottom defined by thickened internodes) were strongly supported as monophyletic (BPP = 1; ML-BS = 100%): *Sporotrichioides*, *Graminearum*, *Novel*, *Sambucinum*, *Longipes*, and *Brachygibbosum*. Putatively novel species resolved within the FSAMSC are designated *F*. sp. nov.-1 to -33. Four phylogenetically distinct species within the *F*. *longipes* Clade previously reported in other studies are identified by unique Arabic numbers (1 to 4). Toxins mapped on the phylogeny were determined via gas chromatography-mass spectrometry. BUT, butenolide; CAL, calonectrin; CUL, culmorin; DAS, diacetoxyscirpenol; NEO, neosolaniol; NIV, nivalenol; T-2, T-2 toxin; ZEA, zearalenone; 3ADON, 3-acetyldeoxynivalenol; 15ADON, 15-acetyldeoxynivalenol; 3OH, isotrichodermol; 3,7-diOH, 7-hydroxy isotrichodermol; 15-decal, 15-decalonectrin; 15-keto NX-2 and 15-keto NX-3, novel trichothecenes; 15-OHCUL, 15-hydroxy culmorin;–, none detected.

Maximum likelihood bootstrap (ML-BS) analyses of the individual *RPB1*, *RPB2*, and *TEF1* partitions were conducted to assess whether they could be analyzed as a combined 3-gene data set. Because trees inferred from the individual genes did not reveal any conflict between strongly supported nodes (S1–S3 Figs), the data sets were combined and analyzed by Bayesian inference (BI) and ML bootstrapping (ML-BS) to assess species boundaries and to gain insight into evolutionary relationships of the 171 strains. The concatenated 3-gene data set included partial *RPB1* (1774 bp alignment, 492 parsimony informative characters = PICs), *RPB2* (1786 bp alignment, 489 PICs), and *TEF1* (640 bp alignment, 276 PICs) gene sequences. Sequences of NRRL 13338 *F*. *nelsonii* from the FCSC, which is sister to the FSAMSC, were selected as the outgroup for rooting the phylogeny. BI and ML analyses of the 3-locus data set yielded similar trees and resolved the 171 strains as 74 phylogenetically distinct species (Fig 1). These included 41 named and 33 putatively novel species, which are informally distinguished here as *F*. sp. nov.-1 to *F*. sp. nov.-33 (Table 1, Fig 1). Analyses of the combined data set revealed that the 39 lineages represented by two or more strains were strongly supported as phylogenetically distinct (BPP = 1; ML-BS = 96–100%, Fig 1). These analyses also resolved the FSAMSC as six strongly supported monophyletic clades (BPP = 1; ML-BS = 100%, Table 1, Fig 1) and evolutionary relationships among them (BPP = 1; ML-BS = 89–100%, Fig 1). The analyses provided moderate to strong support for most nodes (BPP = 0.84–1; ML-BS = 80–100%, Fig 1); however, eight internodes in the interior of the ML phylogeny received bootstrap values < 70% (see *Brachygibbosum*, *Graminearum*, and *Sambucinum* Clades). Four of these internodes, however, were strongly supported by the Bayesian posterior probabilities (BPP = 0.99–1, Fig 1). In addition, seven of the nine internodes that received a BPP < 0.7 (see *Brachygibbosum*, *Graminearum*, *Longipes*, *Sambucinum*, and *Sporotrichioides* Clades) were supported by ML-BS values ≥ 70–95%.

The six multispecies lineages identified within the FSAMSC are (i) the *Sporotrichioides* Clade, which included 32 isolates accounting for 7 described and four novel phylogenetically distinct species closely related to *F*. *armeniacum* (*F*. sp. nov.-1 to -4; Fig 1). Each unnamed putatively novel species was represented by two strains from different geographic origins and isolated from cereals or other hosts/substrates (Table 1). Evolutionary relationships among the four putatively novel species and *F*. *armeniacum* were fully resolved (BPP = 0.99–1; ML-BS = 96–100%, Fig 1); (ii) the *Graminearum* Clade contained one unnamed species (NRRL 34461 *F*. sp. nov.-5) and 22 formally described species, each represented by a single strain (Fig 1). NRRL 34461 *F*. sp. nov.-5, recovered from soil in South Africa, formed a strongly supported cluster with *F*. *acaciae-mearnsii*, *F*. *vorosii*, and *F*. *ussurianum* within what was previously named the *F*. *graminearum* species complex (BPP = 1; ML-BS = 100%, Fig 1). To facilitate communication, given the recent advances in *Fusarium* systematics, we propose abandoning the informal designation *F*. *graminearum* species complex and to refer to this group and closely related species as the *Graminearum* Clade, to emphasize its status as a monophyletic multispecies lineage within the FSAMSC. The *Graminearum* and *Sporotrichioides* Clades were strongly supported as sisters (BPP = 1; ML-BS = 99%, Fig 1); (iii) a *Novel* Clade comprised two phylogenetically distinct species, *F*. sp. nov.-6 and -7 (BPP = 1; ML-BS = 100%, Fig 1). *Fusarium* sp. nov.-6 is represented by three strains: NRRL 54640 and 54683 from Papua New Guinean sweet potato received as *F*. *lateritium* and NRRL 28066 isolated from corn in Japan accessioned as *F*. *graminearum* (S1 Table). *Fusarium* sp. nov.-7 comprised two Ethiopian isolates, NRRL 46743 from wheat seed and NRRL 66736 from soybean plants exhibiting root rot symptoms (S1 Table). BPP and ML bootstrapping resolved the *Novel* Clade as sister to the *Sporotrichioides* + *Graminearum* Clades (BPP = 1; ML-BS = 89%, Fig 1); (iv) the *Sambucinum* Clade included 42 isolates comprising 6 described and 10 putatively novel phylogenetically distinct species (*F*. sp. nov.-8 to -17; Fig 1). The latter were represented by 2 or 3 strains except for *F*. sp. nov.-8, -11, -14, and -17, which are single species lineages (Fig 1). Monophyly of the 6 putatively novel and 4 described species represented by 2 or more strains was strongly supported (BPP = 1; ML-BS = 100%, Fig 1). BPP and ML bootstrapping resolved the *Sambucinum* Clade as a basal sister to the *Sporotrichioides*, *Graminearum*, and *Novel* Clades (BPP = 1; ML-BS = 100%, Fig 1). BI and ML and analyses of the 3-locus data set indicated that the three strains received as *F*. *tumidum* [76] from gorse, lupine, and Scotch broom in New Zealand are *F*. *sambucinum* (S1 Table, Fig 1); (v) the *Longipes* Clade contained 19 isolates that were resolved as 9 phylospecies (BPP = 1; ML-BS = 100%, Fig 1), 5 of which were represented by single strains. Seventeen of the 19 isolates were received as *F*. *longipes* and these included representatives of all 9 phylospecies (S1 Table). Following a previously used informal ad hoc nomenclature, four of the species were distinguished as *F*. *longipes*-1 to -4, and the other 5 as *F*. sp. nov.-18 to -22 (Fig 1). Seven of the 9 species and 15/19 isolates were recovered from soil (Table 1). The *Longipes* Clade was resolved as a basal sister to the *Sporotrichioides*, *Graminearum*, *Novel*, and *Sambucinum* Clades (BPP = 0.91; ML-BS = 94%, Fig 1); and (vi) the *Brachygibbosum* Clade, the earliest diverging clade within the FSAMSC, accounted for 50 of the 171 strains and these included 2 described species (i.e., *F*. *brachygibbosum* and *F*. *transvaalense*) and 11 putatively novel phylogenetically distinct species distinguished as *F*. sp. nov.-23 to -33 (Fig 1). Representatives of 10/13 species and 28/50 of the *Brachygibbosum* Clade isolates were received as *F*. *compactum* (S1 Table). Of the 13 species in this clade, NRRL 66919 *F*. sp. nov.-31 from Ghanaian soybean exhibiting root rot was the only species represented by a single strain (Fig 1). Monophyly of the 12 other species was strongly supported (BPP = 1; ML-BS = 95–100%, Fig 1). Evolutionary relationships within this clade were mostly well-resolved by BPP and ML-BS. However, two pairs of putative sisters were only resolved by the Bayesian analysis, and these are *F*. *transvaalense*–*F*. sp. nov.-31 (BPP = 1; ML-BS = 71%, Fig 1) and *F*. sp. nov.-29–*F*. sp. nov.-30 (BPP = 1; ML-BS = 60%, Fig 1).

### Trichothecene production in broth, rice, and corn substrates

Structurally diverse toxins were produced by the FSAMSC strains in the current study. Of the 171 strains tested 38 failed to synthesize any toxin in the liquid media and solid substates we used (S2 Table). The type A trichothecenes DAS, NEO, and T-2 and type B trichothecenes NIV, 3ADON, and 15ADON were among the most prevalent toxins detected. The six clades resolved by BI and ML bootstrap analyses of the 3-locus data set corresponded to six trichothecene-producing lineages represented by strains that synthesized type A trichothecenes, type B, or both types (S2 Table, Fig 1).

#### *Sporotrichioides* Clade

Within the *Sporotrichioides* Clade, 30/32 strains produced type A trichothecene toxins in vitro. Except for five strains that synthesized DAS, NEO, or T-2 (NRRL 36351 *F*. *nodosum*, NRRL 29896 *F*. sp. nov.-4, NRRL 54940 *F*. *langsethiae*, NRRL 53429 *F*. *sibiricum*, and FRC R-9068 *F*. sp. nov.-3), the other species produced DAS and/or NEO with T-2. Only one strain, FRC R8102 *F*. sp. nov.-1, made the type A trichothecene isotrichodermol (3OH) in agmatine broth. In addition to DAS, NEO, and T-2, the tricyclic sesquiterpene diol culmorin (CUL) was detected in agmatine and YEPD cultures of NRRL 34176 and 36236 *F*. *langsethiae* (S2 Table, Fig 1).

#### *Graminearum* Clade

Of the 23 species in this clade, 17 produced type B trichothecenes, and these included 9 that synthesized NIV (*F*. *acaciae-mearnsii*, *F*. *austroamericanum*, *F*. *brasilicum*, *F*. *cerealis*, *F*. *cortaderiae*, *F*. *gerlachii*, *F*. *meridionale*, *F*. *praegraminearum*, and *F*. *subtropicale*), 4 made 3ADON (*F*. *boothii*, *F*. *pseudograminearum*, *F*. *ussurianum*, and NRRL 34461 *F*. sp. nov.-5), and 4 synthesized 15ADON (*F*. *aethiopicum*, *F*. *graminearum*, *F*. *nepalense*, and *F*. *vorosii*). In addition to NIV, NRRL 31238 *F*. *brasilicum* also produced the type A trichothecene calonectrin (CAL) in agmatine medium. Of the 171 strains included in the present study, NRRL 13393 *F*. *lunulosporum* was the only strain that made the estrogenic mycotoxin zearalenone (ZEA). Toxins were not detected, however, in individual strains of 5 species (*F*. *asiaticum*, *F*. *culmorum*, *F*. *dactylidis*, *F*. *louisianense*, and *F*. *mesoamericanum*) when cultured in liquid media and solid substrates (S2 Table, Fig 1).

#### *Novel* Clade

Two undescribed species within the *Novel* Clade, *F*. sp. nov.-6 and -7, produced type A trichothecenes. Two isolates of *F*. sp. nov.-6 synthesized T-2 and NEO toxins in liquid media. In addition, NRRL 54683 *F*. sp. nov.-6, and the two isolates of *F*. sp. nov.-7, made DAS + NEO + T-2 and NEO + T-2, respectively, in rice cultures (S2 Table, Fig 1).

#### *Sambucinum* Clade

**Fig 2:**
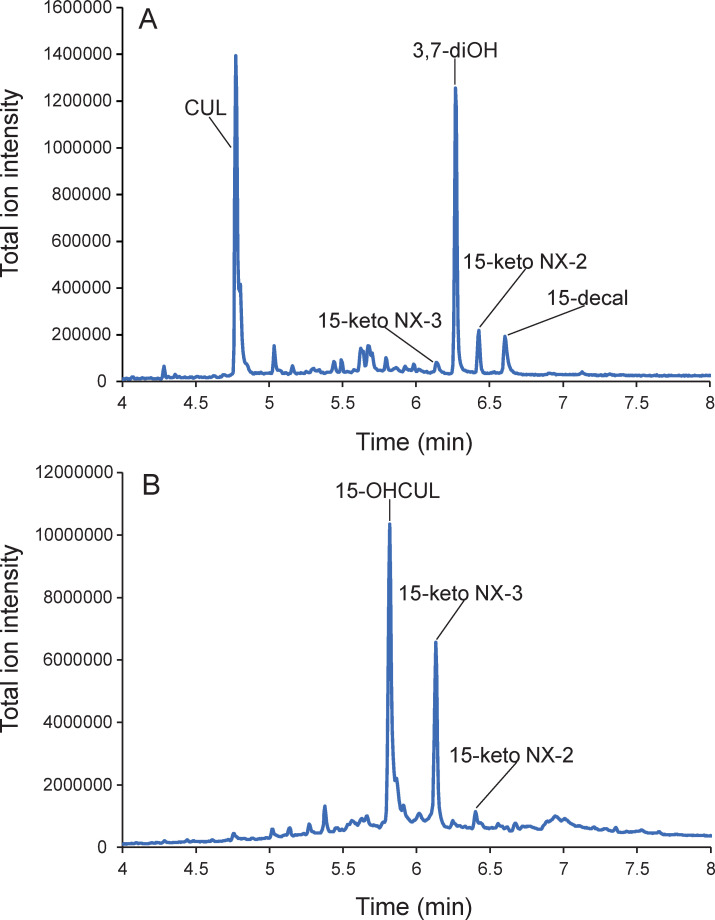
GC-MS analysis of toxic secondary metabolites in vitro. (A) Five toxins were detected in agmatine broth cultures of NRRL 66927 *F*. sp. nov.-16, including the sesquiterpene culmorin (CUL) and four type A trichothecenes. Two of the latter, 7-hydroxy isotrichodermol (3,7-diOH) and 15-decalonectrin (15-decal), were previously known; however, 15-keto NX-2 and its deacetylated form 15-keto NX-3 were first discovered and characterized here. (B) The novel type A trichothecenes 15-keto NX-2 and 15-keto NX-3 were detected in a rice culture of NRRL 66921 *F*. sp. nov.-15, together with 15-hydroxy culmorin (15-OHCUL).

Fourteen of the 16 species within *Sambucinum* Clade only produced type A trichothecenes; however, NRRL 6491 and 3509 *F*. *kyushuense* from Japan synthesized NIV and DAS, which are type B and type A trichothecenes respectively. NRRL 22192 *F*. sp. nov.-11 (= BBA 64918), represented by a single strain from an Indonesian palm tree, was the only species within this clade that failed to produce toxins (Fig 1). In addition to *F*. *kyushuense*, DAS was detected in 9/16 species, T-2 in 7, NEO in 5, CUL in 3, butenolide (BUT) in 3, CAL in 2, and 3OH in 2 (Table 1). Of special note, two putative novel sister species, *F*. sp. nov.-15 and -16, which were received as *F*. *longipes* isolated from South African wheat straw or soil, produced CUL, 7-hydroxy isotrichodermol (3,7-diOH), 15-decalonectrin (15-decal), and two novel type A trichothecenes, 15-keto NX-2 and 15-keto NX-3 in agmatine broth (Fig 2A). The latter two toxins are similar to NX-2 and its deacetylated form NX-3 but differ in that they possess a keto or aldehyde group, respectively, at the C-15 position on the trichothecene ring. When cultured on rice, *F*. sp. nov.-15 and -16 produced the same two novel toxins with 15-hydroxy culmorin (15-OHCUL) (Fig 2B).

#### *Longipes* Clade

Of the 19 isolates comprising 9 phylospecies in the *Longipes* Clade tested, DAS was only produced on rice by one isolate of four different species. The other 15 strains failed to synthesize any toxin in liquid media and on solid substrates (S2 Table, Fig 1).

#### *Brachygibbosum* Clade

The *Brachygibbosum* Clade was represented by 50 isolates comprising 13 phylogenetically distinct species; however, only *F*. *brachygibbosum* and *F*. *transvaalense* possess binomials. Eight of the species synthesized one or more type A trichothecenes (i.e., DAS, NEO, and CAL). Of the 5 remaining species, four produced DAS + NIV, and one only NIV.

In addition to type A and B trichothecenes, several other biologically active secondary metabolites were detected in culture extracts of some isolates in four clades (Table 1, Fig 1). The sesquiterpene CUL was detected in cultures of most strains within the *Graminearum* Clade (14/23), 2 strains in the *Sporotrichioides* Clade, and 6 strains representing 3 novel type A trichothecene-producing species in the *Sambucinum* Clade. In addition, BUT was produced in rice and cornmeal cultures by some isolates of *F*. *venenatum*, NRRL 39685 *F*. sp. nov.-13, and NRRL 26795 *F*. sp. nov.-17 in the *Sambucinum* Clade, and FRC R-4478 *F*. *transvaalense* in the *Brachygibbosum* Clade (S2 Table, Fig 1).

### Pathogenicity test on wheat

**Fig 3:**
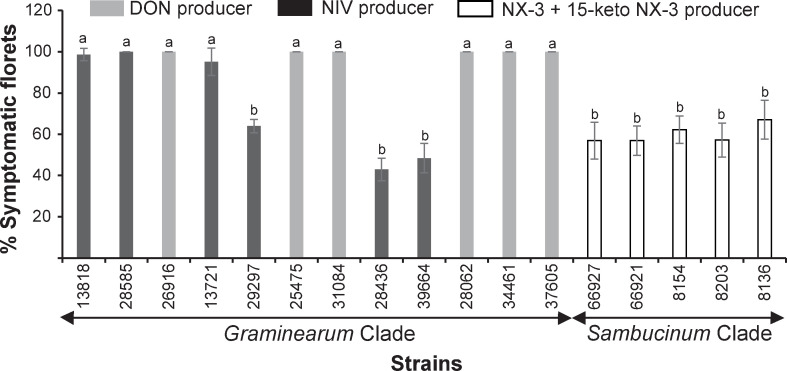
Fusarium head blight spread in Apogee wheat heads inoculated with strains from *Graminearum* and *Sambucinum* Clades. Percentage of symptomatic spikelets (± SEM) was calculated from the average of percent diseased florets in the five inoculated wheat heads 21 days post inoculation. Letters above bars indicate significant differences as determined by ANOVA analysis followed by Fisher’s protected least significant difference (LSD) test for individual comparisons (P < 0.05). Strains represented by four- and five-digit numbers were obtained, respectively, from the FRC and NRRL culture collections (strain identity and history can be found in Table 1).

The pathogenic potential of 75 strains representing the six clades of the FSAMSC (*Brachygibbosum* = 17, *Graminearum* = 12, *Longipes* = 10, *Novel* = 4, *Sambucinum* = 20, and *Sporotrichioides* = 12) was determined by their ability to cause FHB on the highly susceptible wheat cv. Apogee and to spread within the wheat head from a single floret inoculation. Disease spread was evaluated by assessing the percentage of florets exhibiting premature whitening or necrosis at different timepoints, and when the experiment was terminated 21-days post inoculation. Of the 75 strains evaluated, only 17 were nonpathogenic, and heads inoculated with these were asymptomatic and indistinguishable from the negative controls. These nonpathogenic strains were members of the *Brachygibbosum*, *Longipes*, *Novel*, *Sambucinum*, and *Sporotrichioides* Clades. The remaining 58 strains induced FHB symptoms, but the majority (41/58) did not spread beyond the inoculated floret and were classified as nonaggressive (S3 Table). Phenotypic variation in symptoms was noted on wheat heads inoculated with the nonaggressive strains. Some strains produced necrotic lesions or black spots on the inoculated spikelet, while others caused premature bleaching, a typical symptom of FHB. However, bleaching did not spread to neighboring florets. The remaining strains, which belong to the *Graminearum* (n = 12) and *Sambucinum* (n = 5) Clades were able to spread and colonize nearby florets or the entire wheat head. Some of these strains exhibited levels of aggressiveness like the highly aggressive positive control NRRL 31084 *F*. *graminearum* (S3 Table, Fig 3). Strains of *F*. sp. nov.-15 and -16, the two novel species within the *Sambucinum* Clade that produced 15-keto NX-2 and 15-keto NX-3 along with other toxins in culture, induced bleaching on Apogee and spread, causing considerable disease above and below the inoculation point (S4 Table, Fig 3). Variation in aggressiveness was noted among strains tested within the *Graminearum* Clade (S4 Table, Fig 3). NIV-producing species within this Clade (*F*. *austroamericanum*, *F*. *cerealis*, *F*. *cortaderiae*, *F*. *meridionale*, and *F*. *praegraminearum*) displayed moderate (43–64%, Fig 3) to high (95–100%, Fig 3) aggressiveness toward wheat cv. Apogee (Fig 3). Strains of *F*. *cortaderiae*, *F*. *meridionale*, and *F*. *praegraminearum* infected approximately half of the wheat head by the end of the assay, which is modest compared to the more aggressive species *F*. *austroamericanum* and *F*. *cerealis* that were able to spread through the whole head 21 days post inoculation (S4 Table, Fig 3). The 3ADON/15ADON-producing species (*F*. *graminearum*, *F*. *pseudograminearum*, *F*. *vorosii*, *F*. *boothii*, and NRRL 34461 *F*. sp. nov.-5) as well as *F*. *asiaticum* and *F*. *culmorum*, which did not produce toxins in vitro but made NIV and DON, respectively, *in planta* (see below), were able to spread and induce severe head blight symptoms on wheat cv. Apogee where they colonized the entire wheat head 17 to 21 days following single floret inoculation (S4 Table, Fig 3).

### Trichothecene production *in planta*

Analyses of trichothecene production in the inoculated wheat heads showed that two of the 17 nonpathogenic strains (FRC R-738 *F*. *sambucinum* and FRC R-7107 *F*. sp. nov.-12) tested made DAS *in planta*. Similarly, FRC R-8978 *F*. *venenatum*, which was pathogenic but not aggressive produced DAS, 15-monoacetoxyscirpenol, and scirpentriol. Four nonaggressive strains from the *Sporotrichioides* Clade (NRRL 36295 *F*. *sporotrichioides*, FRC R-7479 *F*. *armeniacum*, NRRL 54062 *F*. sp. nov.-2, and NRRL 29296 *F*. sp. nov.-4) synthesized T-2 tetraol 15-acetyl toxin (T-2TE) in the inoculated heads. In addition, NIV was detected in wheat heads inoculated with NRRL 3509 *F*. *kyushuense* from the *Sambucinum* Clade, and NRRL 66919 *F*. sp. nov.-31 and NRRL 66920 *F*. sp. nov.-29 from the *Brachygibbosum* Clade (S3 Table). The other nonaggressive strains in the *Brachygibbosum*, *Longipes*, *Novel*, *Sambucinum*, and *Sporotrichioides* Clades failed to produce any toxin in the inoculated heads (S3 Table). By contrast, the five strains representing *F*. sp. nov.-15 and -16 from the *Sambucinum* Clade, which synthesized 15-keto NX-2 and 15-keto NX-3 along with other toxins in vitro, made NX-3, 15-keto NX-3, and hydroxy culmorin (OHCUL) *in planta* (S3 Table). Analyses of wheat heads inoculated with members of the *Graminearum* Clade revealed that they were all toxigenic *in planta* (S3 Table). Strains of *F*. *austroamericanum*, *F*. *cerealis*, *F*. *cortaderiae*, *F*. *meridionale*, *F*. *praegraminearum*, and *F*. *asiaticum*, which failed to produce any toxin when tested in vitro, made NIV *in planta*. Some of these species also produced CUL and OHCUL (S3 Table). Similarly, CUL and OHCUL were synthesized by *F*. sp. nov.-5, *F*. *culmorum*, and *F*. *pseudograminearum* in the inoculated heads along with deoxynivalenol (DON). The acetyl-ester derivative of DON, 3ADON was also detected in heads inoculated with *F*. *pseudograminearum*. DON was also produced *in planta* by *F*. *boothii*, *F*. *graminearum*, and *F*. *vorosii*.

## Discussion

### Phylogenetic diversity and trichothecene potential of FSAMSC strains

The present study was initiated to investigate species diversity, trichothecene toxin potential, and pathogenicity on wheat within the FSAMSC. Toward this end, 171 FSAMSC strains were selected from the FRC and NRRL culture collections, based on analyses of partial *TEF1* sequence data, to represent the phylogenetic diversity of this species complex. GCPSR-based analysis of combined partial *RPB1*, *RPB2*, and *TEF1* sequence data, together with prior extensive studies on the *Graminearum* Clade [5,6, reviewed in 38], resolved the 171 strains as 74 genealogically exclusive phylospecies distributed among six strongly supported trichothecene toxin-producing clades. This finding is consistent with published genomic data that all FSAMSC species examined to date possess intact trichothecene biosynthetic genes [22,77,78]. However, previous phylogenetic studies have found that the evolutionary history of several *TRI* cluster genes does not mirror the species phylogeny [22,26,78]. As previously reported, results of the current study showed clades within the FSAMSC generally correlate with the type of trichothecene mycotoxin produced [29,49,79,80].

#### *Sporotrichioides* Clade

Consistent with what is known about trichothecene production within this clade (Table 1, [29,48,57,58,60]), the 11 species analyzed, including 4 novel undescribed species closely related to *F*. *armeniacum*, produced DAS, NEO + T-2, or DAS, NEO, and T-2 type A trichothecenes. Toxin production by the 4 newly discovered phylospecies (*F*. sp. nov.-1 to -4) and *F*. *nodosum* is reported here for the first time. The two strains of *F*. *nodosum* studied herein, NRRL 13431 (= FRC T-384) from a tomato stem in Turkey and NRRL 36351 (= CBS 201.63) from stored peanuts in Portugal, were received as *F*. *chlamydosporum*. *Fusarium nodosum* has also been isolated from *Arundo donax* in France and wheat in Iran [8].

#### *Graminearum* Clade

The *Graminearum* Clade isolates investigated here have been the subject of several detailed phylogenetic studies [6,9,10,38,81]. Nineteen of the 23 species in this clade were discovered in prior GCPSR studies over the past two decades and all have been formally described except for NRRL 34461 *F*. sp. nov.-5. The latter species was initially reported as *F*. *acaciae-mearnsii* [35], but subsequently as a putatively novel sister to *F*. *acaciae-mearnsii* [43]. In contrast to *F*. sp. nov.-5 from soil in South Africa, the few characterized strains of *F*. *acaciae-mearnsii* were recovered from *Acacia mearnsii* and wheat in South Africa and soil in Australia [10,35,82]. Prior to the recent discovery of type A trichothecene NX-2 toxin-producing *F*. *graminearum* strains in North America [9,27,28], members of this clade were only known to produce type B trichothecenes. We found that 19/23 strains, each representing a unique phylospecies in this clade, synthesized type B trichothecenes in vitro and/or *in planta*. However, *F*. *brasilicum* made NIV and the type A trichothecene CAL in agmatine broth. Although *F*. *mesoamericanum*, *F*. *louisianense*, and *F*. *dactylidis* failed to synthesize type B trichothecenes here, they are all known to produce them [10,41,43]. Even though NRRL 13393 *F*. *lunulosporum* possesses an intact trichothecene toxin gene cluster [77], it failed to synthesize any trichothecene. However, it did produce zearalenone as previously reported [10]. Global surveys have revealed that *F*. *asiaticum*, *F*. *culmorum*, and *F*. *graminearum* are segregating for 15ADON, 3ADON, and NIV worldwide [26,32,82] whereas the available data indicate the 15ADON chemotype is fixed in *F*. *boothii* [26,82,83]. *Fusarium ussurianum*, by contrast, appears to be fixed for the 3ADON chemotype in the Far East of Russia [44]. Some reports suggest that 3ADON toxin-producing FHB pathogens are more aggressive than those that produce 15ADON on wheat [84]. In North America, an introduced 3ADON population of *F*. *graminearum* has increased in frequency compared to the native 15ADON population [81,84]. These observations raise concerns about the sustainability of FHB-resistant fungicides and emphasize the need for continued monitoring of their population dynamics.

#### *Novel* Clade

The current study also resulted in the discovery of a novel type A T-2, DAS, and NEO-producing trichothecene lineage comprising two novel phylogenetically distinct sister species, *F*. sp. nov.-6 and -7. The former species, *F*. sp. nov.-6, has an Asian-Pacific distribution with isolates from sweet potato in Papua New Guinea and corn in Japan (Table 1). The latter species (i.e., *F*. sp. nov.-7) was recovered from soybean roots exhibiting root rot, a symptom of sudden death syndrome [34], and wheat seed in Ethiopia (Table 1).

#### *Sambucinum* Clade

The ten novel species discovered in this clade were received incorrectly identified as 8 different species (S1 Table). Of the 16 *Sambucinum* Clade species we tested, *F*. *kyushuense* was the only one capable of producing type B NIV and type A DAS trichothecenes, as previously reported [reviewed in 85,86]. Nivalenol and fusarenon-X were first characterized in this species [46,47]. Consistent with previous reports, *F*. *musarum*, *F*. *venenatum*, and *F*. *sambucinum*, including strains of the latter received as *F*. *tumidum* [29,49,87], produced the type A trichothecenes T-2, DAS, and NEO. The same toxins were also synthesized by all but one of the 10 novel species. In addition, two strains of *F*. *venenatum* and NRRL 26795 *F*. sp. nov.-17 (= BBA 70355 received as *F*. *torulosum* from U.S. soil) synthesized CAL. Production of the type A trichothecene 3OH was also detected in cultures of NRRL 22198 *F*. *venenatum* and FRC R-7122 *F*. sp. nov.-12. We found that 2/3 strains of *F*. *poae* synthesized the type A toxin DAS; however, this species has been reported to produce type A and type B trichothecenes, including DAS, NEO, T-2 toxin, and NIV (Table 1, [48]). Proctor et al. [78] discovered that *F*. *poae* lacks the *TRI16* gene, which is required for synthesis of T-2 toxin. Prior to its formal description, the T-2 and HT-2 producer *F*. *langsethiae* was mistakenly thought to be *F*. *poae* [59]. These findings may explain the incorrect reports of T-2 toxin production by *F*. *poae*. *Fusarium venenatum*, the species used to produce Quorn mycoprotein for human consumption, was found to synthesize the select agent DAS when grown in rice culture [29,55]. Consistent with this finding, the isolates of *F*. *venenatum* analyzed here all produced DAS, but some also made CAL, 3OH, and BUT in agmatine broth and on rice. The Quorn production strain NRRL 26139 (= IMI 145425 = ATCC 20334 = A 3/5; [88]), however, failed to produce DAS and any other toxin in agmatine and rice cultures [McCormick, unpubl, 14,55]. Given that a comparative genomic analysis, which included the Quorn production strain ATCC 20334 revealed it possesses the genes required for type A trichothecene biosynthesis [89], the open question is: Why aren’t they produced in agmatine medium? Agmatine is a strong inducer of *TRI5*, the gene that encodes the first enzymatic step in the trichothecene biosynthesis pathway [73]. When the Quorn production strain A3/5 (= ATCC 20334) was originally isolated in 1968 [88] it was identified as *F*. *graminearum* [90]; however, this species was not formally recognized as *F*. *venenatum* until 1995 [91]. The present study failed to recover additional strains of *F*. *musarum* [92], the earliest diverging lineage within the *Sambucinum* Clade. Similarly, *F*. *robustum* NRRL 13392 recovered from a rotten stem of Paraná pine (*Araucaria angustifolia*) in Argentina [93] is only represented by the ex-type strain.

Global surveys indicated that NX-2 toxin-producing strains of *F*. *graminearum* are restricted to Canada and the upper Midwest and Northeast U.S., suggesting that this chemotype might be indigenous to this region [27,28,94]. Thus, the discovery of two species within the *Sambucinum* Clade from South Africa that produced novel type A trichothecenes in vitro similar to NX-2 and NX-3, but with a keto or aldehyde moiety at the C-15 position, and NX-3, the deacetylated form of NX-2 *in planta* is one of the most important findings of the present study. This discovery is the first to document that NX-2 toxin-producing fusaria are present outside of North America. Although these species were recovered from soil and debris, future studies are needed to determine whether these novel toxins contaminate food and feed and pose a threat to food safety and human health.

#### *Longipes* Clade

Previous phylogenetic studies have showed that the morphospecies *F*. *longipes* encompasses at least four phylospecies [67,95]. Our analyses of 19 strains from the FRC and NRRL culture collections indicate this clade comprises at least 9 phylogenetically distinct species. The fact that only 4/19 strains representing four species produced DAS in rice, but not in agmatine, YEPD, and cornmeal media, suggests the experimental conditions used may have been suboptimal, or these soil inhabiting fusaria may have lost the ability to synthesize trichothecenes. To our knowledge beauvericin production by NRRL 13368 *F*. *longipes*-1 is the only toxin report from this clade [45]. However, a recent phylogenomic study revealed the presence of an intact *TRI* cluster in the genome of NRRL 20695 *F*. *longipes*-4; but trichothecene production by this strain was not investigated [77].

#### *Brachygibbosum* Clade

Our analysis of 50 strains within this clade resolved 13 phylospecies, including two named (i.e., *F*. *brachygibbosum* and *F*. *transvaalense*; [7,96]), and 11 unnamed putatively novel phylospecies. Two of the strains, NRRL 13829 *F*. sp. nov.-23 (= MRC 2568) from river sediment in Japan and NRRL 31008 *F*. *transvaalense* (= BBA 63772) from soil in Australia were previously reported as *F*. cf. *compactum* and *F*. *brachygibbosum*, respectively [67,94]. Although NRRL 13829 was recently reported as *F*. *brachygibbosum* [7], by including the ex-type strain of *F*. *brachygibbosum* NRRL 20954 (= BBA 64691) from Indian sorghum [96], the present phylogenetic analysis resolved these two strains as phylogenetically distinct sister taxa. All 13 species produced trichothecenes, and these included NRRL 66937 *F*. sp. nov.-30 from pearl millet in Botswana that made the type B toxin NIV, 8 type A producers, including *F*. *brachygibbosum* NRRL 20954, and four species that produced type A and B trichothecenes, including *F*. *transvaalense*.

Several strains in each of the six clades failed to produce trichothecenes in vitro under the experimental conditions used. For example, strains of *F*. *asiaticum*, *F*. *louisianense*, and *F*. *dactylidis* tested failed to synthesize any trichothecene in the current study, but they have been shown to synthesize NIV [11,41,43]. Therefore, this failure to produce trichothecenes might be because some strains have lost their toxigenic potential because they have been in culture for decades or the growth conditions we employed were suboptimal for trichothecene production for some strains. The latter hypothesis is supported by studies that found trichothecene production on agmatine was influenced by strain diversity [29,73]. Further research is required to identify optimal nutrient profiling conditions to stimulate trichothecene production in vitro and/or *in planta*. This is supported by the fact that some strains that failed to produce toxins in agmatine broth, which is a strong inductor of trichothecene synthesis [73], made trichothecenes when grown on rice or cornmeal (S2 Table).

The sesquiterpene diol culmorin was produced by most of the species within the *Graminearum* Clade, three novel species within the *Sambucinum* Clade, and by *F*. *langsethiae* in the *Sporotrichioides* Clade. Biosynthesis of this toxin requires a terpene synthase (TS) and a cytochrome P450 monooxygenase encoded by *CLM1* and *CLM2*, respectively [97,98]. A comparative phylogenomic analysis revealed that the two genes were conserved and were present in the genomes of *TRI* cluster-containing species closely related to *F*. *graminearum* within the FSAMSC [77]. By contrast, one or both genes were absent in the more distantly related species *F*. *sambucinum*, *F*. *poae*, *F*. *sporotrichioides*, *F*. *longipes*, and *F*. *langsethiae* [77]. Thus, the fact that CUL was detected in *F*. *langsethiae* herein and in previous studies [48,59] suggests the genomes should be screened for the presence of these two genes, given that intraspecific variation in the presence and absence of certain secondary metabolite gene clusters was previously noted [99].

Overall, the BI and ML analyses revealed 101 of the 171 strains included in the present study were received misidentified, in some instances under names that are not accepted today (i.e., *F*. *trichothecioides* and *F*. *sulphureum* = *F*. *sambucinum*). It is worth mentioning that most of the species within the *Graminearum* Clade (n = 19) were also received misidentified, mostly as *F*. *graminearum*, prior to GCPSR studies of this clade [10,38]. The present phylogenetic analysis also revealed that close to half of the genealogically exclusive lineages (33/74 = 44.6%) represented novel phylospecies. In addition to the 23 *Graminearum* Clade strains, each representing a unique phylospecies, the other 148 strains were resolved as 19 previously described and 32 novel phylospecies in the following five FSAMSC clades: *Brachygibbosum* (n = 50 strains, 11/13 novel species), *Sambucinum* (n = 42 strains, 10/16 novel species), *Sporotrichioides* (n = 32 strains, 4/11 novel species), *Longipes* (n = 19 strains, 5/9 novel species), and a *Novel* Clade (n = 5 strains, 2/2 novel species). The current study highlights the importance of multilocus DNA sequence typing (MLST) as an essential tool for accurately identifying fusaria. To illustrate their impact, studies over the past two decades indicate that *Fusarium* comprises several fold more species than included in the traditional morphology-based taxonomic treatments [100]. For example, in a reassessment of fusaria included in Marasas et al. [51], a MLST analysis [29] found that the 17 morphospecies included in this compendium comprised 46 phylospecies, including 10 that were putatively novel.

### Pathogenicity on wheat and trichothecene production *in planta*

We evaluated pathogenicity, aggressiveness, and trichothecene production of 75 strains representing 57 species spanning six clades of the FSAMSC on wheat (cv. Apogee). Our data showed that only members of the *Graminearum* Clade and two novel species within the *Sambucinum* Clade were aggressive. Strains of the other species were either not pathogenic to wheat or caused head blight symptoms in the inoculated floret without spreading to nearby spikelets. Furthermore, most of these strains failed to produce detectable levels of mycotoxins in the inoculated wheat heads. The failure of these strains to cause FHB and to spread may be because they did not produce trichothecenes, which are required for fungal spread and FHB development in some Triticeae [17,19]. The nonaggressive strains belong to five clades other than *Graminearum* and most were isolated from non-gramineous hosts or other substrates. This finding suggests that the FSAMSC may contain nonpathogenic species that possess an endophytic or saprophytic lifestyle. For example, while *F*. *poae* and *F*. *sporotrichioides* are widely recovered from cereals, they are less aggressive secondary invaders of heads infected by the major FHB pathogen *F*. *graminearum* [101]. The available data also suggest that *F*. *armeniacum*, *F*. *brachygibbosum*, *F*. *longipes*, and *F*. *venenatum* have evolved principally as common saprophytes of diverse crops, natural grasses, soil, and other substrates [102]. All strains within the *Longipes* Clade and more than half in the *Brachygibbosum* Clade were recovered from soil and debris. By contrast, *Sporotrichioides* and *Sambucinum* Clade strains were recovered from a wide range of hosts including cereals, wild grasses, soil, and debris.

A detailed comparative genomics study of *F*. *venenatum* and *F*. *graminearum* revealed a high degree of synteny between the two genomes, including genes known to be involved in virulence [89]. *Fusarium venenatum* may be nonpathogenic to wheat because it is missing two transcription factors previously shown to be required for *F*. *graminearum* virulence on wheat and five *F*. *graminearum* specific secondary metabolite gene clusters that are typically upregulated during infection of wheat by *F*. *graminearum*. The combined loss of these genes, and the toxigenic difference between DON-producing *F*. *graminearum* and DAS-producing *F*. *venenatum*, are thought to contribute to the lack of aggressiveness toward wheat in *F*. *venenatum* and the divergent lifestyles of these fungi [89]. These findings also highlight the need for further research on the evolution of pathogenesis in other species within the FSAMSC to better understand what factors contribute to FHB pathogen aggressiveness toward cereals.

In that regard, variation in aggressiveness was observed among the *Graminearum* Clade strains tested in the current study (Fig 3). The NIV-producing species *F*. *austroamericanum*, *F*. *asiaticum*, and *F*. *cerealis* were highly aggressive on wheat (cv. Apogee) and spread throughout most of the head at 21-days post inoculation. However, other NIV-producing species were less aggressive (*F*. *cortaderiae*, *F*. *meridionale*, and *F*. *praegraminearum*). Our data are consistent with other reports that evaluated these species on other wheat cultivars including cv. Norm [6,11,41,83]. Even so, toxin analyses of the inoculated wheat heads revealed that these species were all toxigenic and made NIV *in planta*. *Graminearum* Clade strains that produced 3ADON or 15ADON in vitro displayed high aggressiveness on wheat and were able to spread throughout the entire wheat heads. Tracking disease progress at different timepoints revealed that these species rapidly spread and colonized the entire heads 17 days post inoculation. Consistent with their ability to spread throughout the wheat head, GC-MS analyses showed they produced DON *in planta*. Overall, these findings match previous reports that indicate DON functions as a virulence factor allowing the fungus to overcome wheat defenses and spread throughout wheat heads [17,19].

In addition to producing type B trichothecenes, most of the *Graminearum* Clade strains tested produced CUL and/or its derivative OHCUL along with DON or NIV *in planta*. Culmorin is considered an “emerging mycotoxin” that is frequently found in naturally contaminated grain and axenic cultures of the most important FHB causal agents where it co-occurs with DON and other trichothecene analogs [103]. However, information on the toxicological relevance of CUL is limited. Nevertheless, a recent study demonstrated that CUL and DON have a synergistic phytotoxic effect on cereals when the concentration of CUL exceeded that of DON [77]. In addition, these authors reported that the severity of FHB induced by *F*. *graminearum* on wheat was positively correlated with the sum of CUL and DON. Given that CUL inhibits the activity of UDP-glucosyltransferases [104], their working hypothesis is CUL suppresses glycosylation of DON by serving as an alternate substrate for UDP-glucosyltransferases [77]. While 15 of the 20 *Sambucinum* Clade isolates tested for pathogenicity on wheat were not aggressive, strains of the novel species *F*. sp. nov.-15 and -16 induced FHB and showed levels of aggressiveness on cv. Apogee comparable to members of the *Graminearum* Clade (Fig 3). Mycotoxin analyses revealed that these newly discovered sister species produced the novel toxins 15-keto NX-2 and 15-keto NX-3 with other toxins in vitro and 15-keto NX-3, NX-3, and the sesquiterpene OHCUL *in planta*. Future research is required to better understand the pathophysiological impact of these novel toxins and to determine whether they function as virulence factors against wheat and other small grain cereals. Results of the pathogenicity assay provide evidence that the potential to induce FHB and aggressiveness are clearly influenced by the type of toxin produced and this appears to be a strain rather than a clade-specific characteristic. *In planta* toxin data showed that type B trichothecene NIV- and DON-producing strains from the *Graminearum* Clade and the type A trichothecene NX-3 and 15-keto NX-3 producers from the *Sambucinum* Clade were aggressive toward wheat. By contrast, NIV producers from the *Sambucinum* and *Brachygibbosum* Clades and strains from other clades, which synthesized toxins such as DAS and T-2TE toxin, were unable to spread in the inoculated wheat heads. While the disease assays herein were only conducted on cv. Apogee, selected for its short life cycle that made it feasible to screen such a large strain subset, the data obtained provide robust genotype-pathogenic phenotype correlations between diverse strains within the FSAMSC. Furthermore, these data provide a framework for subsequent investigations on other cultivars with different degrees of susceptibility to FHB, which will be needed to further elucidate the role of toxins on pathogen aggressiveness, including the novel trichothecenes.

To summarize, phylogenetic, biochemical, and pathogenicity analyses have provided insights into the phylogenetic diversity, trichothecene toxin potential, and pathogenicity to wheat within the FSAMSC. The robust MLST scheme employed, combined with toxin analyses and pathogenicity assays led to several important discoveries (i) the FSAMSC comprises at least 74 genealogically exclusive phylospecies, 33 of which are novel, distributed among six trichothecene toxin-producing clades; (ii) a *Novel* type A trichothecene-producing clade comprising two sister species; (iii) two putatively novel species within the *Sambucinum* Clade from South Africa produced NX-3 and two novel type A trichothecenes similar to NX-2 and NX-3 but with a keto or aldehyde group, respectively, at the C-15 position on the trichothecene ring; and (iv) only members of the *Graminearum* Clade and *F*. sp. nov.-15 and -16 within the *Sambucinum* Clade were aggressive on cv. Apogee, which suggests that aggressiveness is likely determined by the type of toxin produced. These results clearly illustrate that robust molecular systematic data are essential for accurately identifying species within the FSAMSC and they provide a firm foundation for ongoing comparative phylogenomic analyses of these agronomically important fusaria, which are focused on advancing global agricultural biosecurity, plant health and food safety.

## Supporting information

S1 Fig Molecular phylogeny of the *Fusarium sambucinum* species complex (FSAMSC) inferred from partial *RPB1* sequences of 171 isolates using MrBayes 3.2.7a and IQ-TREE 1.6.12. The phylogeny was rooted on a sequence of NRRL 13338 *F*. *nelsonii* from the *F*. *chlamydosporum* species complex. Support values above or below branches are Bayesian posterior probabilities (BPP)/maximum likelihood bootstrap values (ML-BS). Thickened black internodes identify six strongly supported clades (BPP = 1; ML-BS = 98–100%): *Brachygibbosum*, *Graminearum*, *Longipes*, *Novel*, *Sambucinum*, and *Sporotrichioides*. The FSAMSC comprises 74 phylogenetic species, including 33 that appear to be novel (i.e., *F*. sp. nov.-1 to -33).
(TIF)

S2 Fig Molecular phylogeny of the *Fusarium sambucinum* species complex (FSAMSC) inferred from partial *RPB2* sequences of 171 isolates using MrBayes 3.2.7a and IQ-TREE 1.6.12. The phylogeny was rooted on a sequence of NRRL 13338 *F*. *nelsonii* from the *F*. *chlamydosporum* species complex. Support values above or below branches are Bayesian posterior probabilities (BPP)/maximum likelihood bootstrap values (ML-BS). Six clades of the FSAMSC were strongly supported as monophyletic (BPP = 1; ML-BS = 95–100%): *Brachygibbosum*, *Graminearum*, *Longipes*, *Novel*, *Sambucinum*, and *Sporotrichioides*. The FSAMSC comprises 74 phylogenetic species, including 33 that appear to be novel (i.e., *F*. sp. nov.-1 to -33).
(TIF)

S3 Fig Molecular phylogeny of the *Fusarium sambucinum* species complex (FSAMSC) inferred from partial *TEF1* sequences of 171 isolates using MrBayes 3.2.7a and IQ-TREE 1.6.12. The phylogeny was rooted on a sequence of NRRL 13338 *F*. *nelsonii* from the *F*. *chlamydosporum* species complex. Support values above or below branches are Bayesian posterior probabilities (BPP)/maximum likelihood bootstrap values (ML-BS). Six clades of the FSAMSC were strongly supported as monophyletic (BPP = 1; ML-BS = 95–100%): *Brachygibbosum*, *Graminearum*, *Longipes*, *Novel*, *Sambucinum*, and *Sporotrichioides*. The FSAMSC comprises 74 phylogenetic species, including 33 that appear to be novel (i.e., *F*. sp. nov.-1 to -33).
(TIF)

S1 Table Strain histories. (XLSX)

S2 Table Toxins produced by *Fusarium sambucinum* species complex strains in liquid media, rice grain, and cornmeal. (XLSX)

S3 Table Pathogenicity of *Fusarium sambucinum* species complex strains on cv. Apogee and toxin production *in planta*. (XLSX)

S4 Table Spread of Fusarium head blight symptoms on cv. Apogee after inoculation with strains belonging to *Graminearum* and *Sambucinum* Clades. (XLSX)
