# Correction: Phylogenetic diversity, trichothecene potential, and pathogenicity within *Fusarium sambucinum* species complex

**DOI:** 10.1371/journal.pone.0250812

**Published:** 2021-04-22

**Authors:** Imane Laraba, Susan P. McCormick, Martha M. Vaughan, David M. Geiser, Kerry O’Donnell

Fig 1 in the original article [1] is incorrect. Fig 1 should have been individual figures, rather than one consolidated figure. The authors have provided correct versions of Fig 1 as new figures below. Figs 4–7 correspond with the originally published Fig 1. Please view Figs 4–7 here.

**Fig 4:**
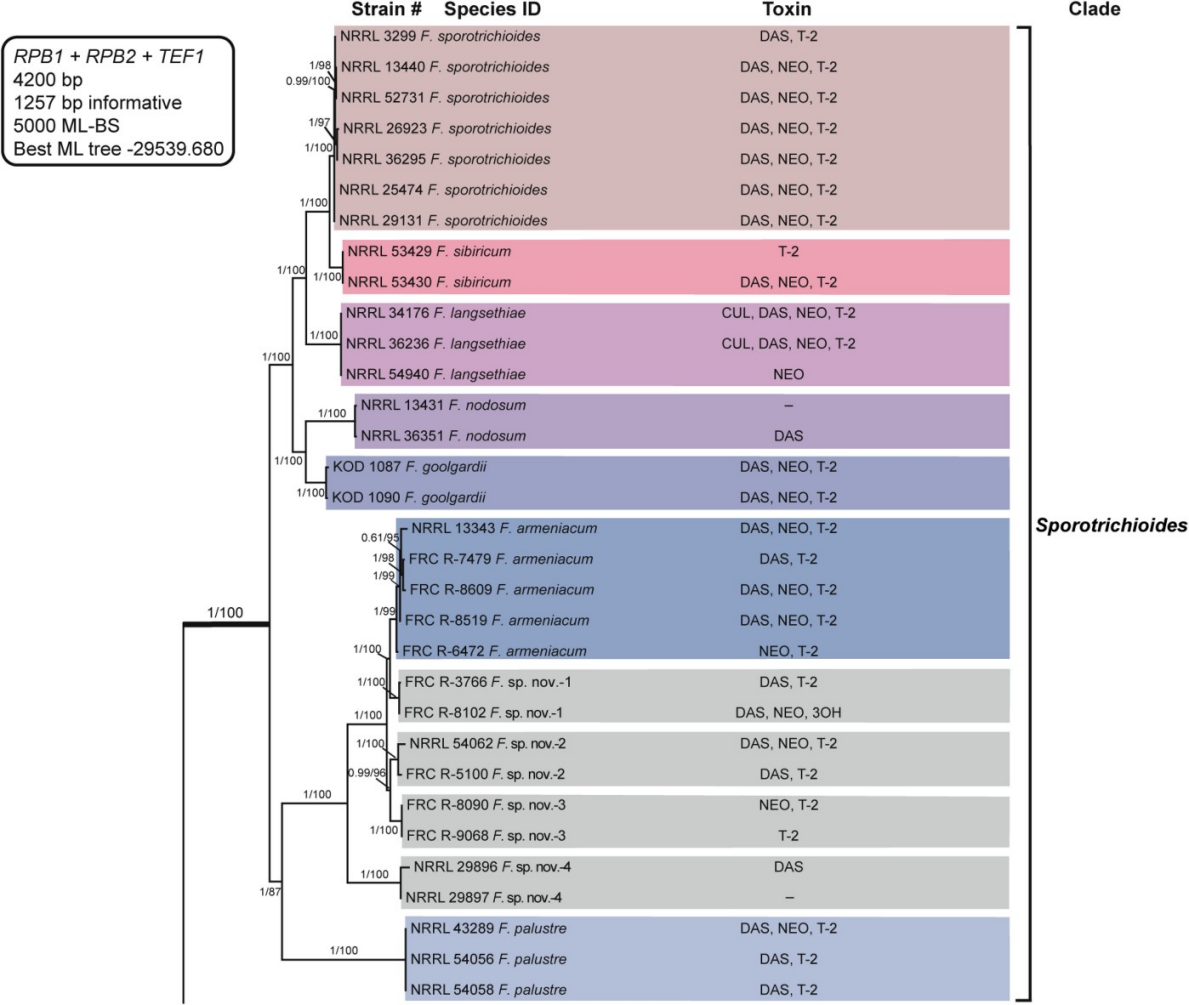
*Fusarium sambucinum* species complex. Bayesian and maximum likelihood phylogeny of the *Sporotrichioides* Clade inferred from partial *RPB1* + *RPB2* + *TEF1* data set. Support values above internodes are Bayesian posterior probabilities (BPP)/maximum likelihood bootstrap (ML-BS) values. BPP were calculated using MrBayes 3.2.7a [71]. ML-BS values were determined using IQ-TREE 1.6.12 [66]. The *Sporotrichioides* Clade (defined by thickened internode) was strongly supported as monophyletic (BPP = 1; ML-BS = 100%). Putatively novel species resolved within the *Sporotrichioides* Clade are designated *F*. sp. nov.-1 to -4. Toxins mapped on the phylogeny were determined via gas chromatography-mass spectrometry. CUL, culmorin; DAS, diacetoxyscirpenol; NEO, neosolaniol; T-2, T-2 toxin; 3OH, isotrichodermol;–, none detected.

**Fig 5:**
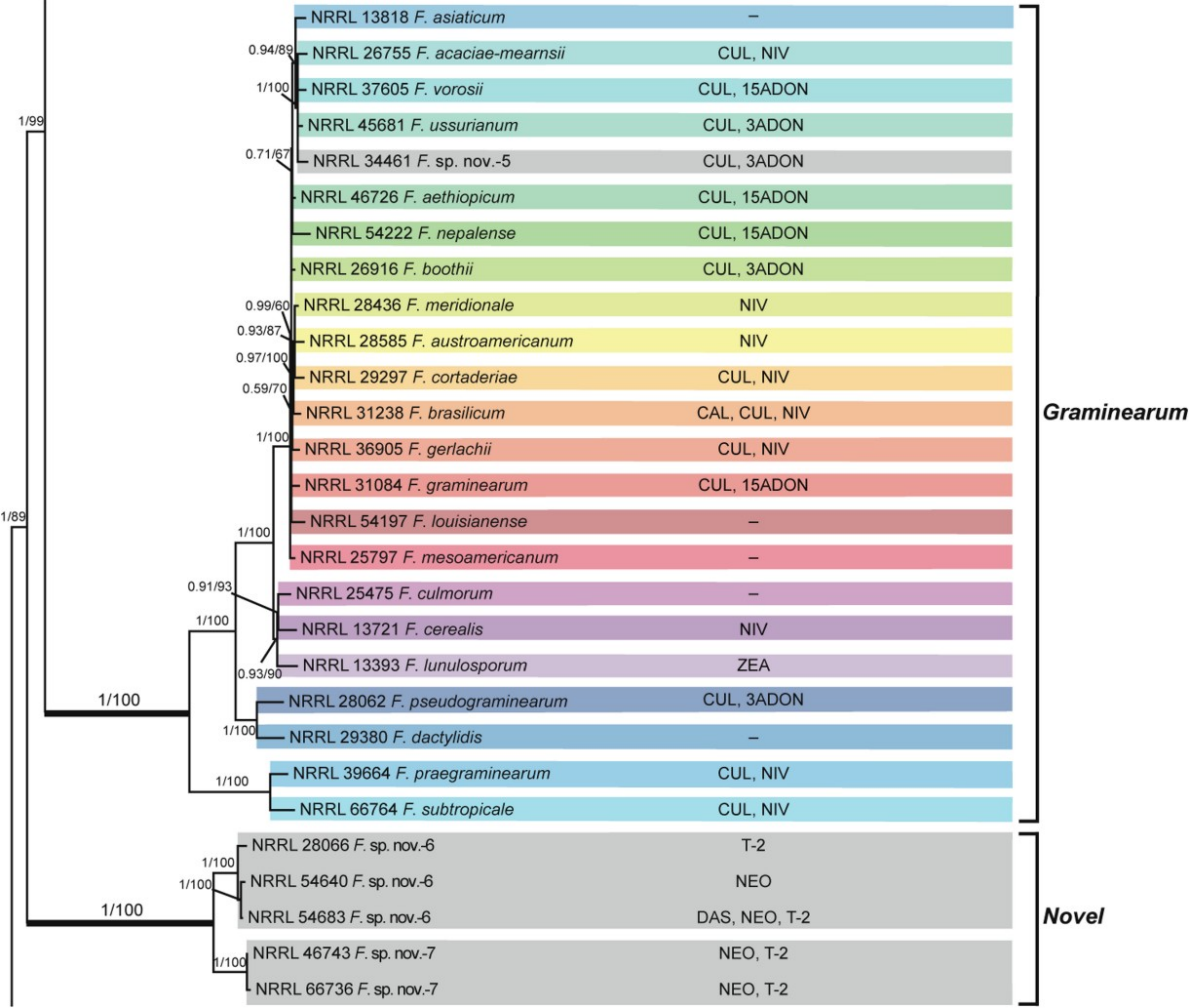
*Fusarium sambucinum* species complex. Bayesian and maximum likelihood phylogeny of the *Graminearum* and *Novel* Clades inferred from partial *RPB1* + *RPB2* + *TEF1* data set. Support values above internodes are Bayesian posterior probabilities (BPP)/maximum likelihood bootstrap (ML-BS) values. BPP were calculated using MrBayes 3.2.7a [71]. ML-BS values were determined using IQ-TREE 1.6.12 [66]. The *Graminearum* and *Novel* Clades (defined by thickened internodes) were strongly supported as monophyletic (BPP = 1; ML-BS = 100%). Putatively novel species resolved within the *Graminearum* and *Novel* Clades are designated *F*. sp. nov.-5 to -7. Toxins mapped on the phylogeny were determined via gas chromatography-mass spectrometry. CAL, calonectrin; CUL, culmorin; DAS, diacetoxyscirpenol; NEO, neosolaniol; NIV, nivalenol; T-2, T-2 toxin; ZEA, zearalenone; 3ADON, 3-acetyldeoxynivalenol; 15ADON, 15-acetyldeoxynivalenol;–, none detected.

**Fig 6:**
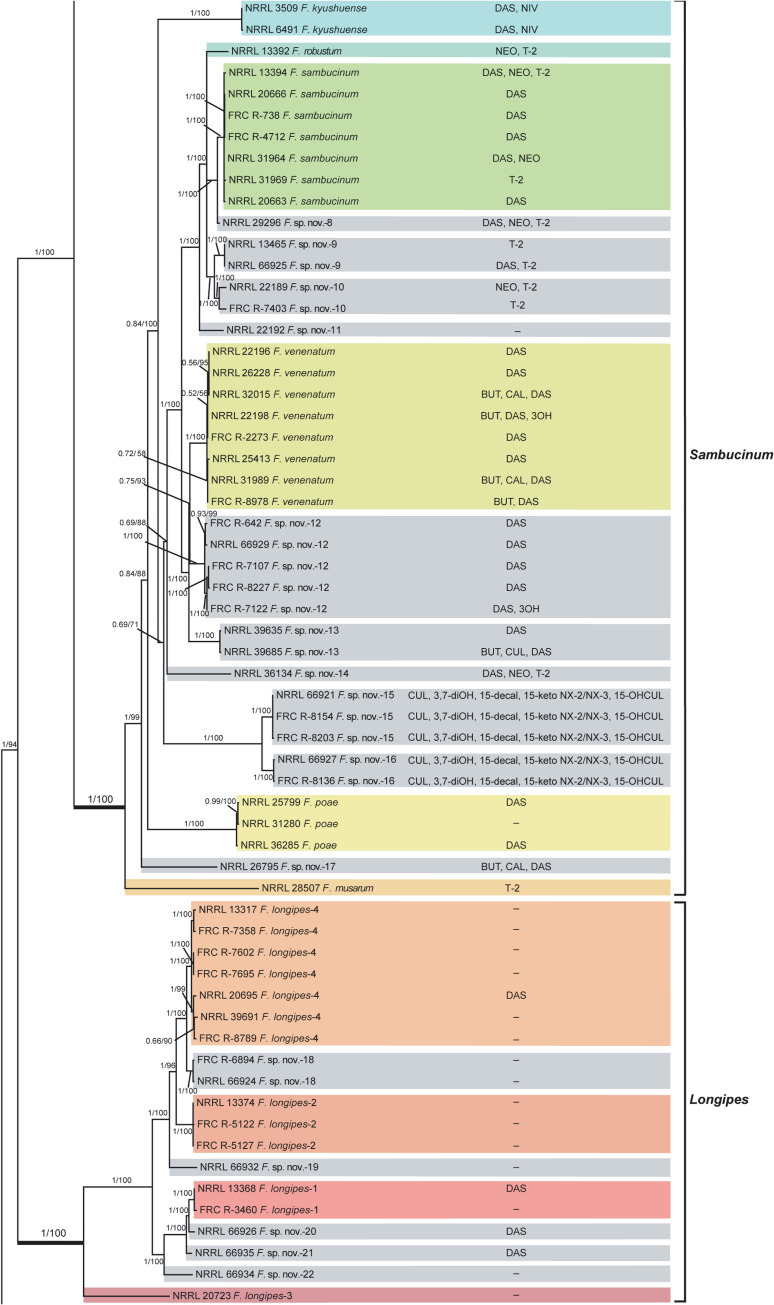
*Fusarium sambucinum* species complex. Bayesian and maximum likelihood phylogeny of the *Sambucinum* and *Longipes* Clades inferred from partial *RPB1* + *RPB2* + *TEF1* data set. Support values above internodes are Bayesian posterior probabilities (BPP)/maximum likelihood bootstrap (ML-BS) values. BPP were calculated using MrBayes 3.2.7a [71]. ML-BS values were determined using IQ-TREE 1.6.12 [66]. The *Sambucinum* and *Longipes* Clades (defined by thickened internodes) were strongly supported as monophyletic (BPP = 1; ML-BS = 100%). Putatively novel species resolved within the *Sambucinum* and *Longipes* clades are designated *F*. sp. nov.-8 to -22. Four phylogenetically distinct species within the *Longipes* Clade previously reported in other studies are identified by unique Arabic numbers (1 to 4). Toxins mapped on the phylogeny were determined via gas chromatography-mass spectrometry. BUT, butenolide; CAL, calonectrin; CUL, culmorin; DAS, diacetoxyscirpenol; NEO, neosolaniol; NIV, nivalenol; T-2, T-2 toxin; 3OH, isotrichodermol; 3,7-diOH, 7-hydroxy isotrichodermol; 15-decal, 15-decalonectrin; 15-keto NX-2 and 15-keto NX-3, novel type A trichothecenes; 15-OHCUL, 15-hydroxy culmorin;–, none detected.

**Fig 7:**
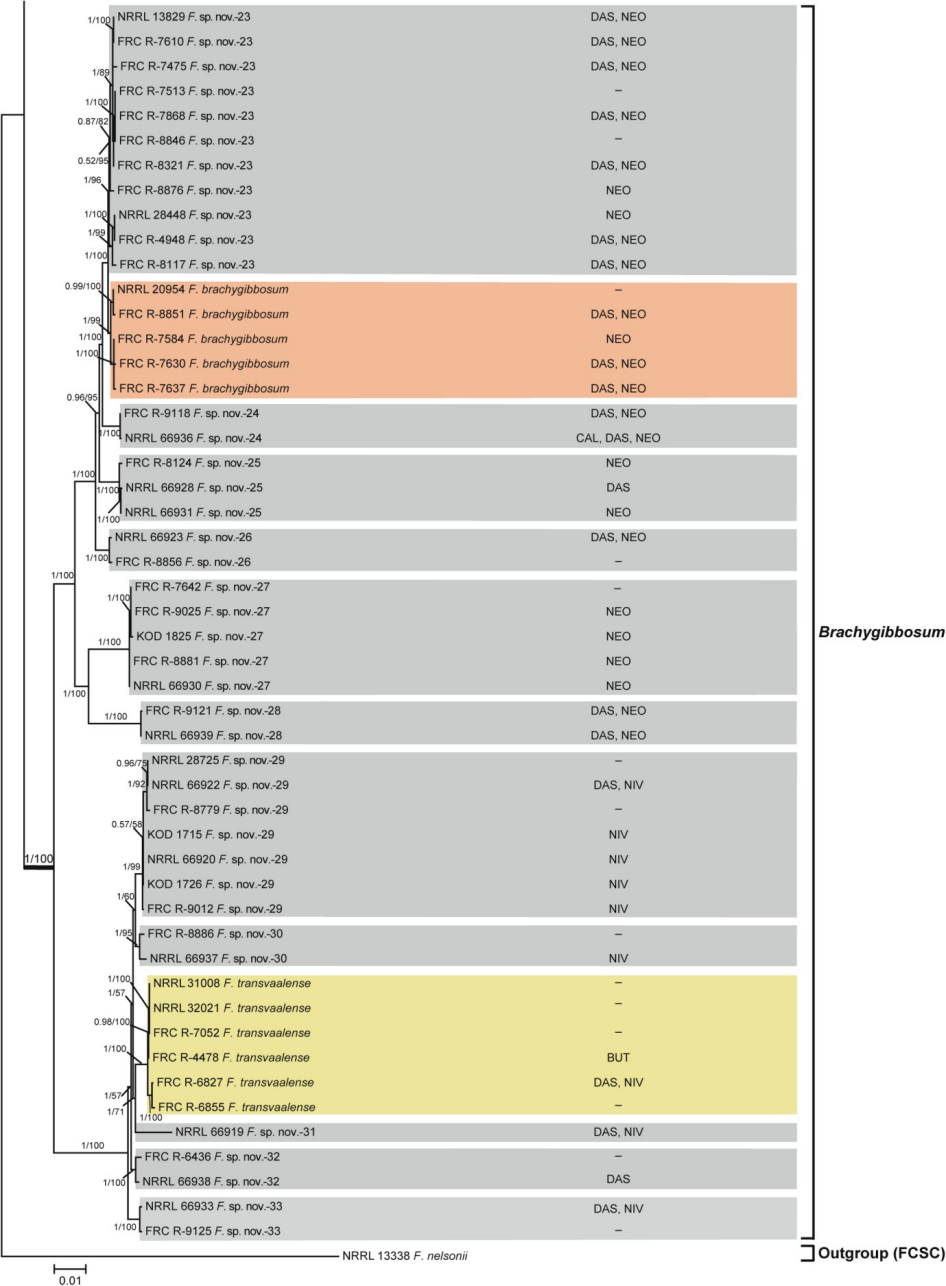
*Fusarium sambucinum* species complex. Bayesian and maximum likelihood phylogeny of the *Brachygibbosum* Clade inferred from partial *RPB1* + *RPB2* + *TEF1* data set. Support values above internodes are Bayesian posterior probabilities (BPP)/maximum likelihood bootstrap (ML-BS) values. BPP were calculated using MrBayes 3.2.7a [71]. ML-BS values were determined using IQ-TREE 1.6.12 [66]. The ingroup *Fusarium sambucinum* species complex was rooted on sequences of NRRL 13338 *F*. *nelsonii* from its sister group, the *F*. *chlamydosporum* species complex. The *Brachygibbosum* Clade (defined by thickened internode) was strongly supported as monophyletic (BPP = 1; ML-BS = 100%). Putatively novel species resolved within the *Brachygibbosum* Clade are designated *F*. sp. nov.-23 to -33. Toxins mapped on the phylogeny were determined via gas chromatography-mass spectrometry. BUT, butenolide; CAL, calonectrin; DAS, diacetoxyscirpenol; NEO, neosolaniol; NIV, nivalenol;–, none detected.
